# Assessing Global Water Storage Variability from GRACE: Trends, Seasonal Cycle, Subseasonal Anomalies and Extremes

**DOI:** 10.1007/s10712-016-9367-1

**Published:** 2016-02-26

**Authors:** Vincent Humphrey, Lukas Gudmundsson, Sonia I. Seneviratne

**Affiliations:** Institute for Atmospheric and Climate Science, ETH Zurich, Universitaetstrasse 16, 8092 Zurich, Switzerland

**Keywords:** GRACE, Water storage, Precipitation, Temperature, Drought, Signal decomposition

## Abstract

Throughout the past decade, the Gravity Recovery and Climate Experiment (GRACE) has given an unprecedented view on global variations in terrestrial water storage. While an increasing number of case studies have provided a rich overview on regional analyses, a global assessment on the dominant features of GRACE variability is still lacking. To address this, we survey key features of temporal variability in the GRACE record by decomposing gridded time series of monthly equivalent water height into linear trends, inter-annual, seasonal, and subseasonal (intra-annual) components. We provide an overview of the relative importance and spatial distribution of these components globally. A correlation analysis with precipitation and temperature reveals that both the inter-annual and subseasonal anomalies are tightly related to fluctuations in the atmospheric forcing. As a novelty, we show that for large regions of the world high-frequency anomalies in the monthly GRACE signal, which have been partly interpreted as noise, can be statistically reconstructed from daily precipitation once an adequate averaging filter is applied. This filter integrates the temporally decaying contribution of precipitation to the storage changes in any given month, including earlier precipitation. Finally, we also survey extreme dry anomalies in the GRACE record and relate them to documented drought events. This global assessment sets regional studies in a broader context and reveals phenomena that had not been documented so far.

## Introduction

Land water resources are essential for human society and are affected by climate variability and human water use (Jiménez Cisneros et al. 2014). It is thus important to monitor changes in land water storage, as well as the underlying processes leading to their variations in space and time. The Gravity Recovery and Climate Experiment (GRACE), launched in 2002, constitutes an essential tool for such analyses, as was demonstrated in a wealth of studies (Tapley et al. 2004a; Wahr et al. 2004; Rodell et al. 2004; Andersen et al. 2005; Velicogna and Wahr 2006; Güntner et al. 2007a; Ramillien et al. 2008; Zaitchik et al. 2008; Rodell et al. 2009; Chen et al. 2010a; Houborg et al. 2012; Sasgen et al. 2012; Gardner et al. 2013; Wouters et al. 2014; Reager et al. 2014; Famiglietti 2014; Chen et al. 2015; Wahr 2015). After more than a decade of observations, the GRACE mission has resulted in an unprecedented view on global water storage variability, with a great diversity in terms of temporal scales, ranging from long-term trends to short-lived deviations from the seasonal cycle. These different scales of temporal variability often constitute a common denominator between GRACE studies, either implicitly—as when the discussion focuses on specific aspects like the seasonal cycle, trends or extremes—or explicitly—as when water storage time series are decomposed into subseries. Since the earliest GRACE studies, it has been, for instance, very common to refer to the phasing and amplitude of the seasonal cycle when comparing GRACE terrestrial water storage with other datasets such as model simulations (e.g., Tapley et al. 2004b; Wahr et al. 2004). As the temporal coverage of the GRACE record extended, more comprehensive studies also identified secular trends and inter-annual anomalies by separating the GRACE signal into long-term trends, periodical components and residual noise (Ramillien et al. 2005; Schmidt et al. 2008b; Steffen et al. 2009). However, there is still no global overview on the relative magnitude and distribution of these features of temporal variability. In addition, while certain of these features (e.g., seasonal cycles and trends) are relatively well described, others (e.g., high-frequency residuals and extremes) have typically attracted much less attention so far and remain more difficult to interpret.

From a global perspective, terrestrial water storage anomalies derived from GRACE are dominated by a seasonal signal in most parts of the world. Consequently, the earliest studies comparing GRACE with hydrological models have primarily focused on the seasonal component. Most often, the seasonal cycle in GRACE was shown to compare relatively well with model simulations, both with respect to the signal’s amplitude and phase (Wahr et al. 2004; Swenson and Milly 2006; Syed et al. 2008; Schmidt et al. 2008b; Döll et al. 2014a). Reviews (Ramillien et al. 2008; Güntner 2008; Schmidt et al. 2008a) showed that seasonal disagreement between GRACE and model data was usually attributed to deficiencies in the modelling of water storage compartments and to errors in the precipitation forcing, but also to signal leakage and inaccuracies of the GRACE data. Multiple studies have shown that long-term variability in the GRACE record over land can be related to long-term trends in groundwater (Rodell et al. 2009; Voss et al. 2013; Döll et al. 2014b; Chen et al. 2015; Richey et al. 2015a, b) and surface water (Swenson and Wahr 2009; Singh et al. 2012), teleconnections (Phillips et al. 2012) and mass variations in the cryosphere (Sasgen et al. 2012; Velicogna and Wahr 2013). The hydrological signal extracted from GRACE can also be contaminated by glacial isostatic adjustment (Wu et al. 2010) and crustal deformations caused by major earthquakes (Han et al. 2006, 2011, 2013). While the seasonal cycle, long-term anomalies and secular trends are arguably well documented, fewer studies have focused on subseasonal variability and extreme events at a global scale. So far only case studies have shown that major drought and flood events can be observed in the GRACE record (e.g., Andersen et al. 2005; Seitz et al. 2008; Frappart et al. 2012; Long et al. 2013; Abelen et al. 2015). Only recently, the potential of GRACE for monitoring drought conditions (Houborg et al. 2012; Thomas et al. 2014) and predicting flood potential (Reager et al. 2014) was investigated globally. However, large challenges remain since month-to-month variability in GRACE is highly contaminated with outliers, measurement errors and uncertainties arising from data processing (Bonin et al. 2012).

The overarching goal of this study is to provide a global and comprehensive survey of the dominant features of temporal variability in terrestrial water storage observed from GRACE. Our approach is to decompose the total signal at each grid point into (1) *linear trends*, (2) *inter*-*annual variability*, (3) *seasonal cycle* and (4) *subseasonal variability*. We first assess the contribution of each component to the total signal at the global scale (Sect. 4.1). In Sect. 4.2, the magnitude and significance of the linear trends are discussed in the context of previous regional studies. Subsequently, the decomposed subseries of terrestrial water storage are compared with decomposed precipitation and temperature fields. Starting with the inter-annual anomalies, regions of high correlation between GRACE and these atmospheric drivers are identified (Sect. 4.3). Section 4.4 provides global maps of the maximum and minimum seasonal water storage and identifies phase shifts with respect to the seasonal cycles of both precipitation and temperature (Sect. 4.4). In Sect. 4.5, we focus on the subseasonal residuals and show that a careful averaging of the daily atmospheric data to the monthly resolution reveals excellent correlations with the high-frequency component of the GRACE signal. Finally, we use the decomposition approach to identify and analyse drought events in the GRACE record (Sect. 4.6).

## Data

### GRACE Data

**Fig. 1:**
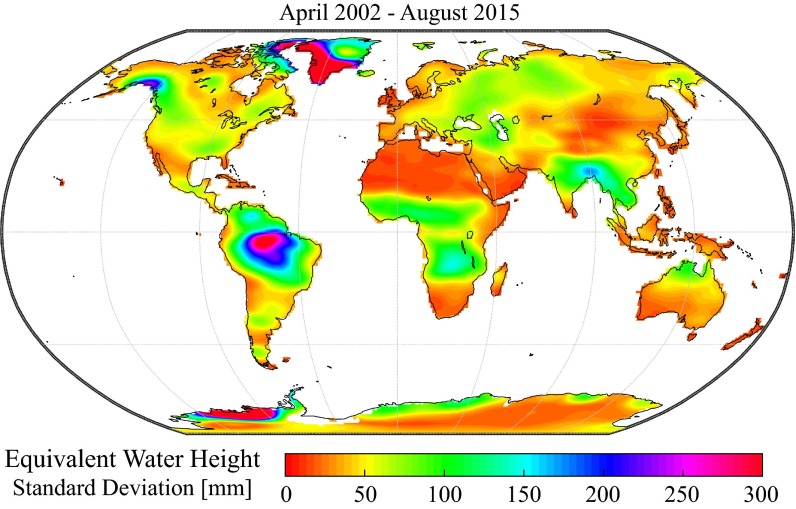
Standard deviation of equivalent water height from the ensemble mean of GRACE Tellus CSR, JPL and GFZ solutions (mm)

Monthly grids of terrestrial water storage anomalies used in this study are based on the spherical harmonic coefficients (Release 05) provided by the Center for Space Research (CSR), the Jet Propulsion Laboratory (JPL) and the GeoForschungsZentrum Potsdam (GFZ) for the period April 2002–August 2015. For more information on the GRACE mission, the gravity recovery process and the derivation of water storage anomalies from the spherical harmonic coefficients, we refer the reader to the reviews from Wouters et al. (2014) or Wahr (2015) and the references therein. The gridded product used in this study is the GRACE Tellus dataset (available at http://grace.jpl.nasa.gov). This dataset provides mass grids in units of equivalent water height for the three different sets of harmonic coefficients mentioned above, at a temporal resolution of approximately 1 month and with a grid resolution of 1°. It is worth noting that although 1° (or even finer) grids are commonly used in global analyses of terrestrial water storage anomalies, this does not reflect the actual spatial resolution of the GRACE measurements. Due to the truncation of spherical harmonics, the effective spatial resolution is by construction limited to a few hundreds of kilometres (Landerer and Swenson 2012). Additionally, postprocessing filters that are used to reduce spatially correlated errors further degrade the spatial resolution of the GRACE signal (Swenson and Wahr 2006; Duan et al. 2009; Longuevergne et al. 2010; Frappart et al. 2011b; Wouters et al. 2014). This causes spatial autocorrelation in the gridded dataset, as can be seen in Fig. 1, which also provides a general overview of the regions where hydrological variability, as detected by GRACE, has the largest magnitude.

At the time of writing, the GRACE Tellus product is obtained through the following processing: the degree one harmonic coefficients (Earth’s geocenter) are estimated from Swenson et al. (2008), the coefficients of degree-order 2–0 (related to Earth oblateness) are replaced with more reliable solutions from Satellite Laser Ranging (Cheng et al. 2011) and correction for glacial isostatic adjustment is applied following Geruo et al. (2013). A known issue is that GRACE maps are heavily contaminated with correlated noise; hence, several spatial filtering techniques have been proposed that aim at restoring the geophysical signal (Kusche 2007; Ramillien et al. 2008; Werth et al. 2009; Frappart and Ramillien 2012). In the Tellus product, the destriping filter of Swenson and Wahr (2006) is applied to correct for North–South oriented stripes in GRACE maps and a 300 km Gaussian filter is additionally applied to the data to reduce residual noise. Finally, it is worth mentioning that GRACE time series are not evenly spaced in time. GRACE “months” most often do not correspond to calendar months due to instrument issues and solutions for several months can be missing, in particular after 2011. Instead, GRACE months represent approximately 1 month long periods with varying numbers of days.

Sources of errors in GRACE include measurement errors, aliasing errors originating from the inaccurate correction of atmospheric and oceanic mass redistribution, and spatial leakage (Seo et al. 2006). Spatial leakage is caused both by the truncation of spherical harmonics and the postprocessing filters applied to the data (Chen et al. 2007a; Landerer and Swenson 2012). Since there are no other large-scale observations of terrestrial water storage that could be used as ground truth, estimating errors and confidence intervals for GRACE data is a major challenge (Güntner 2008). One possibility to reduce uncertainty in the GRACE data is to use the ensemble mean of the solutions obtained by different processing centres (Werth et al. 2009; Sakumura et al. 2014). In this study, we use the mean of the three solutions from CSR, JPL and GFZ provided in the GRACE Tellus dataset.

In order to correct for the amplitude attenuation caused by the postprocessing filters applied to the GRACE data, the Tellus dataset also provides the scaling factors proposed by Landerer and Swenson (2012). These scaling factors are derived by first applying the complete GRACE processing to modelled estimates of terrestrial water storage and subsequently comparing the agreement between the original and processed model data. A disadvantage of these scaling factors is that they can depend on the hydrological model used as a reference, especially in semi-arid and arid regions as well as over irrigated areas (Long et al. 2015). Long et al. (2015) also mention that scaling factors found in some regions should be interpreted carefully. For these reasons, scaling factors were not applied to the GRACE data in this study.

### Filtered Grids of Atmospheric Reanalysis

The atmospheric reanalysis ERA-Interim, from the European Centre for Medium-Range Weather Forecasts (ECMWF), is used to derive daily fields of mean 2 m air temperature and precipitation totals (Dee et al. 2011; available at http://apps.ecmwf.int/datasets/data/interim-full-daily/). This dataset is obtained at a 0.25° resolution and averaged to the 1° resolution of the GRACE Tellus dataset. However, the effective spatial resolution of the hydrological signal observed in GRACE is still coarser than 1°, due to the resolution of the GRACE measurements (see Sect. 2.1). For the Tellus product, this effective spatial resolution is approximately 300 km (3° at the equator). In order to make the atmospheric data comparable with GRACE, we apply a 300 km Gaussian filter to the atmospheric grids. Without this filter, the atmospheric fields would show much more detailed patterns than the GRACE data. It is important to note that when GRACE solutions are compared with modelled estimates of terrestrial water storage, a common practice is to apply the whole GRACE processing to the model data, including an expansion of the modelled mass distribution into spherical harmonics and the subsequent postprocessing (e.g., Wahr et al. 2004; Schmidt et al. 2006; Swenson and Milly 2006; Syed et al. 2008). However, this latter approach cannot be applied to global fields of temperature and precipitation, which is why we only apply a Gaussian filter. We also note that the correlations between GRACE and filtered atmospheric fields are expected to increase as a consequence of this filtering. This effect has already been documented in a similar setting by Abelen and Seitz (2013) when comparing GRACE results with both modelled and remotely sensed soil moisture.

## Methods

### Signal Decomposition

#### Background and Previous Approaches

Decomposition of the GRACE hydrological signal is common practice in the recent literature, and different methods have been used to address different objectives. One possibility is to aim at isolating the contribution of specific water storage compartments such as groundwater, soil moisture or snow mass to the total GRACE signal. This leads to highly underdetermined inversion problems of blind signal separation and gives rise to non-unique solutions as the contributing geophysical signals are most often not statistically independent. To account for this, inversion methods have been proposed that can use higher-order statistical information derived from model data to decompose the total signal (Ramillien et al. 2004, 2005; Frappart et al. 2006; Schmeer et al. 2012). Assimilation of GRACE data into a land surface scheme could also be seen as another approach relating GRACE variability to water storage compartments that are already partitioned in a model structure (Zaitchik et al. 2008; Eicker et al. 2014). In groundwater studies, a common strategy is to directly subtract model estimates of snow storage, soil moisture and surface water from the total GRACE signal and use the remainder as an estimate of groundwater changes (Rodell and Famiglietti 2002; Rodell et al. 2007, 2009; Chen et al. 2015). Another decomposition approach is based on extracting the dominant *spatio*-*temporal* patterns of long-term trends and periodic GRACE signals by means of dimensionality reduction methods. This has been done, for instance, with principal component analysis (Schrama et al. 2007; Rangelova et al. 2007; Schmidt et al. 2008b), independent component analysis (Forootan and Kusche 2012; Frappart et al. 2011b) or multichannel singular spectrum analysis (Rangelova et al. 2010). A last option is based on extracting *temporal* components (i.e. at each grid cell) using time series decomposition techniques. This approach has been used to assess the properties and the relative importance of the resulting features of temporal variability (Barletta et al. 2012; Frappart et al. 2013). Occasionally, the employed decomposition also assumes that the data follows a predefined pattern, as, for instance, when the seasonal cycle is represented by fitted harmonic functions (Steffen et al. 2009). In this paper, we aim at a temporal decomposition of the time series, making as few assumptions as possible and accounting for the irregular spacing of the GRACE “months”. This additive decomposition is summarized in Eq. 1, where the original signal (*X*_tot_) is represented as the sum of a *long*-*term component* (*X*_long_), a *seasonal cycle* (*X*_seas_) and the remaining *subseasonal residuals* (*X*_sub_). These high-frequency residuals are expected to be a combination of both a real signal representing subseasonal water storage variability and the noise that is present in the GRACE data. The *long*-*term component* (*X*_long_) is further divided into *linear trends* (*X*_lin_) and the anomalies with respect to this linear trend, being here referred to as *inter*-*annual variability* (*X*_inter_).

1 $$X_{\text{tot}} = \underbrace {{X_{\text{long}} }}_{{X_{\text{lin}} + X_{\text{inter}} }} + X_{\text{seas}} + X_{\text{sub}}$$

#### Seasonal Trend Decomposition Using Loess (STL)

The Seasonal Trend Decomposition using Loess procedure (STL) introduced by Cleveland et al. (1990) is a robust decomposition method that is used to extract the mean seasonal cycle and to separate the remaining deseasonalized signal into a low- and a high-frequency component, where the low-frequency component should contain only periodicities larger than 12 months. This procedure was already used with GRACE data by Baur (2012) and Hassan and Jin (2014) as a method to derive the long-term component, in Bergmann et al. (2012) to robustly deseasonalize GRACE time series, and in Frappart et al. (2013) to compare terrestrial water storage with monthly rainfall time series in the Amazon basin. It has also been successfully applied, for instance, in a hydro-climatological setting (Gudmundsson et al. 2011) or to extract temperature trends (Dufresne et al. 2013). The STL procedure is based on locally weighted smoothing of the deseasonalized time series in which the smoothing parameters are analytically optimized to minimize spectral leakage between the high- and the low-frequency components. We introduce here an adaptation of the original algorithm allowing us to apply this method to unevenly spaced time series, accounting for the irregular temporal spacing of the GRACE data. The STL procedure consists of passes of different smoothing filters and includes the calculation of robustness weights in order to account for the possible influence of outliers in the time series. A detailed description of the modified algorithm is presented in Appendix 1.

**Fig. 2:**
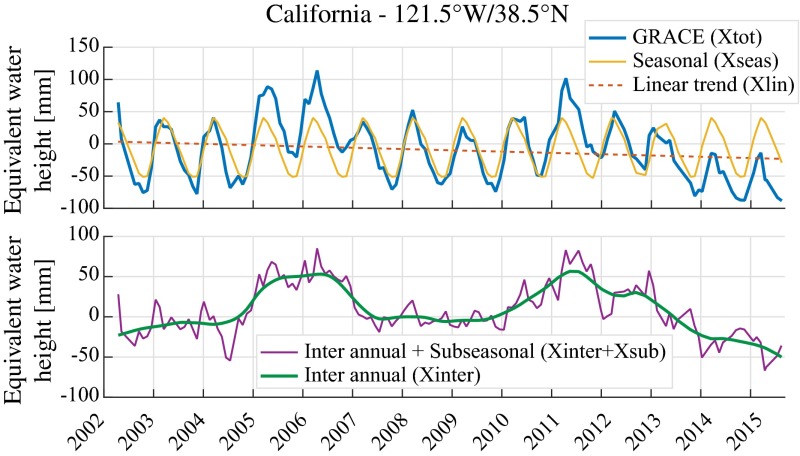
Example of signal decomposition (see Eq. 1) at a grid cell located in California

The STL procedure decomposes the time series into the three components: *X*_seas_, *X*_sub_ and *X*_long_ (Eq. 1). The latter component *X*_long_ is the long-term (or low-frequency) component of the time series and is further decomposed into the components *X*_lin_ and *X*_inter_ (Eq. 1). The linear trend *X*_lin_ is first estimated from the long-term component (*X*_long_) using the Theil–Sen estimator (Sen 1968), and *X*_inter_ is computed as the deviation from this linear trend (*X*_inter_ = *X*_long_ − *X*_lin_). Compared to classical linear regression, using the Theil–Sen slope provides an estimate of the trend that is more robust and less sensitive to large anomalies occurring near the beginning or the end of the time series. This procedure is applied to each grid cell of both the *monthly* GRACE data and the *daily* atmospheric forcing so that we obtain decomposed time series for each of these datasets. In Fig. 2, we illustrate how the presented approach decomposes the GRACE signal into the different subcomponents for the case of a specific grid cell located in California.

### Monthly Averaging of the Daily Decomposed Forcing Time Series

The decomposed daily atmospheric forcing data need to be averaged to monthly values in order to enable a comparison with the GRACE time series. The common approach for this is to use the monthly arithmetic mean (e.g., Frappart et al. 2013; Forootan et al. 2014a; Ahmed et al. 2014). As a reference method, we use the arithmetic mean of the days exactly covered by each GRACE monthly solution. We thus obtain monthly series for each component of the atmospheric daily series. In addition, we present hereafter a more sophisticated averaging method that accounts for storage processes that specifically influence the high-frequency component (*X*_sub_).

#### Limitations of the Arithmetic Mean for the Comparison of High-Frequency Anomalies

When comparing averaged time series of water storage with precipitation, some systematic errors are introduced simply because of the arbitrarily chosen averaging intervals (e.g., monthly intervals in the present case). As water storage is a state and precipitation a flux variable, temporal averages can at times cause a mismatch of the two monthly time series, especially in the case of high-frequency anomalies. A typical example is when a very large precipitation event occurs just at the end of a given month: this extreme event will have a large effect on the precipitation average of the given month but its influence on water storage will be most relevant for the subsequent months. Such artefacts are often falsely attributed to observational errors. In order to address this issue, we propose an alternative to the arithmetic mean that takes the effect of earlier precipitation into account.

#### Comparing Flux and State Variables at Different Temporal Resolutions

**Fig. 3:**
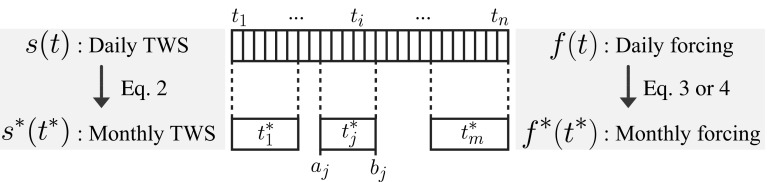
Schematic representation of the correspondence between daily time series and the irregular—quasi-monthly—temporal resolution of the GRACE time series. For daily time steps, $t = \left\{ {t_{1} , \ldots ,t_{i} , \ldots ,t_{n} } \right\}$ going from the 1st to the *n*th day. For quasi-monthly time steps, $t^{*} = \left\{ {t_{1}^{*} , \ldots ,t_{j}^{*} , \ldots ,t_{m}^{*} } \right\}$ going from the 1st to the *m*th month, $a_{j}$ and $b_{j}$ denoting the first and last days of a given month

Hereafter, precipitation anomalies correspond to a time-dependent *flux* variable, denoted $f\left( t \right)$ where $t = \left\{ {t_{1} , \ldots ,t_{i} , \ldots ,t_{n} } \right\}$ is an evenly spaced time vector of length *n*, with units of days. Similarly, daily water storage anomalies correspond to a time-dependent *state* variable denoted $s\left( t \right)$. In our case, the *state* variable $s\left( t \right)$ is not observed at the daily time scale; however, the GRACE product provides *average values* of $s\left( t \right)$ for arbitrary time intervals which approximately correspond to a month. We define this new averaged time series as $s^{*} \left( {t^{*} } \right)$, where $t^{*} = \left\{ {t_{1}^{*} , \ldots ,t_{j}^{*} , \ldots ,t_{m}^{*} } \right\}$ is an unevenly spaced time vector of length *m* corresponding to the GRACE “months”. The relation between $s\left( t \right)$ and $s^{*} \left( {t^{*} } \right)$ can be represented by the arithmetic mean (see Fig. 3 for a schematic illustration of the presented relations):

2 $$s^{*} \left( {t_{j}^{*} } \right) = \frac{1}{{n_{j} }}\mathop \sum \limits_{{t_{i} \in \left[ {a_{j} ,b_{j} } \right]}} s\left( {t_{i} } \right)$$

where $a_{j}$ and $b_{j}$ correspond to the edges of the *j*th time interval (e.g., of the *j*th GRACE month) and $n_{j}$ is the number of days falling within this interval ($n_{j} = b_{j} - a_{j}$).

Our main concern is now to determine the relation between $f\left( t \right)$ and $f^{*} \left( {t^{*} } \right)$. As mentioned above, a common approach is to compute the mean of the daily values over the given time intervals. Analogously to Eq. 2, this simply corresponds to:

3 $$f^{*} \left( {t_{j}^{*} } \right) = \frac{1}{{n_{j} }}\mathop \sum \limits_{{t_{i} \in \left[ {a_{j} ,b_{j} } \right]}} f\left( {t_{i} } \right)$$

Here, we suggest the use of a weighted mean of $f\left( t \right)$ as an alternative approach:

4 $$f^{*} \left( {t_{j}^{*} } \right) = \mathop \sum \limits_{i = 1}^{n} \hat{W}\left( {t_{j}^{*} ,t_{i} } \right) \cdot f\left( {t_{i} } \right)$$

where the normalized weights $\hat{W}\left( {t_{j}^{*} ,t_{i} } \right)$, which will be defined in the next section, depend both on $t_{j}^{*}$ and $t_{i}$ and have the property that:

5 $$\mathop \sum \limits_{i = 1}^{n} \hat{W}\left( {t_{j}^{*} ,t_{i} } \right) = 1$$

#### Weights Based on Integrated Exponential Decay Functions

A simple way to represent the effect of a short-term precipitation anomaly (e.g., a daily precipitation event) on the subsequent state of water storage is the exponential decay function. This is equivalent to assuming linear storage components (bucket models), which is common practice in conceptual hydrological modelling (Beven 2012). Here, we assume that the influence of a flux anomaly (e.g., a precipitation event) on the state variable (e.g., water storage) will decrease exponentially with time. Formally, we define $w\left( {t,t_{i} } \right)$ as the influence of a given flux anomaly $f\left( {t_{i} } \right)$ observed at time $t_{i}$ on the subsequent values of the state variable $s\left( t \right)$ at time $t > t_{i}$.

6 $$w\left( {t,t_{i} } \right) = \left\{ {\begin{array}{*{20}c} {0,\quad {\text{if}} \quad t < t_{i} } \\ {{\text{e}}^{{ - \frac{1}{\tau }\left( {t - t_{i} } \right)}} , \quad {\text{if}} \quad t \ge t_{i} } \\ \end{array} } \right.$$

where $\tau$ is a free parameter controlling the rate of exponential decay and is expressed in units of time (e.g., in days). The influence of the given flux anomaly $f\left( {t_{i} } \right)$ on the earlier values of the state variable (i.e. when $t < t_{i}$) is of course zero.

However, $w\left( {t,t_{i} } \right)$ only represents the influence of $f\left( {t_{i} } \right)$ on the subsequent daily values of $s\left( t \right)$, but we are in fact interested in the influence of $f\left( {t_{i} } \right)$ on the values of $s^{*} \left( {t^{*} } \right)$—the monthly values. For a given $t_{j}^{*}$, summing $w\left( {t,t_{i} } \right)$ over the corresponding time interval $t \in \left[ {a_{j} ,b_{j} } \right]$ yields:

7 $$W\left( {t_{j}^{*} ,t_{i} } \right) = \mathop \sum \limits_{{t \in \left[ {a_{j} ,b_{j} } \right]}} w\left( {t,t_{i} } \right)$$

**Fig. 4:**
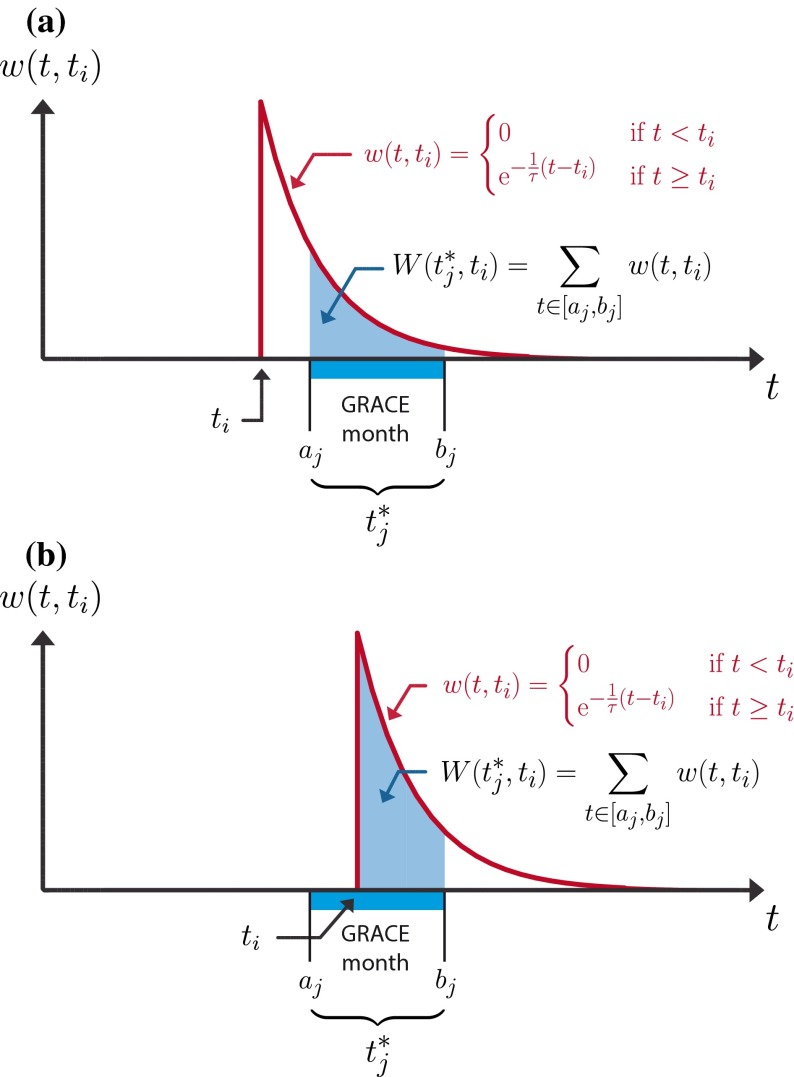
Illustration of Eqs. 6 (*red*) and 7 (*blue*). The red curve depicts the exponentially decaying influence of a given daily flux anomaly (precipitation) occurring at time *t*_i_ on the state variable (water storage) at subsequent time steps. The summation of this influence over the interval covered by a given GRACE month corresponds to the relative weight (*blue area*) assigned to the flux at time *t*_i_. See the text for a description of the different symbols

For illustrative purposes, this summation is shown in Fig. 4. The two examples correspond to the case of a flux anomaly $f\left( {t_{i} } \right)$ occurring either before (Fig. 4a) or during (Fig. 4b) the given time interval $\left[ {a_{j} ,b_{j} } \right]$. The last step is to ensure that the property set by Eq. 5 is fulfilled by normalizing the weights (Eq. 8):

8 $$\hat{W}\left( {t_{j}^{*} ,t_{i} } \right) = \frac{{W\left( {t_{j}^{*} ,t_{i} } \right)}}{{\mathop \sum \nolimits_{i = 1}^{n} W\left( {t_{j}^{*} ,t_{i} } \right)}}$$

**Fig. 5:**
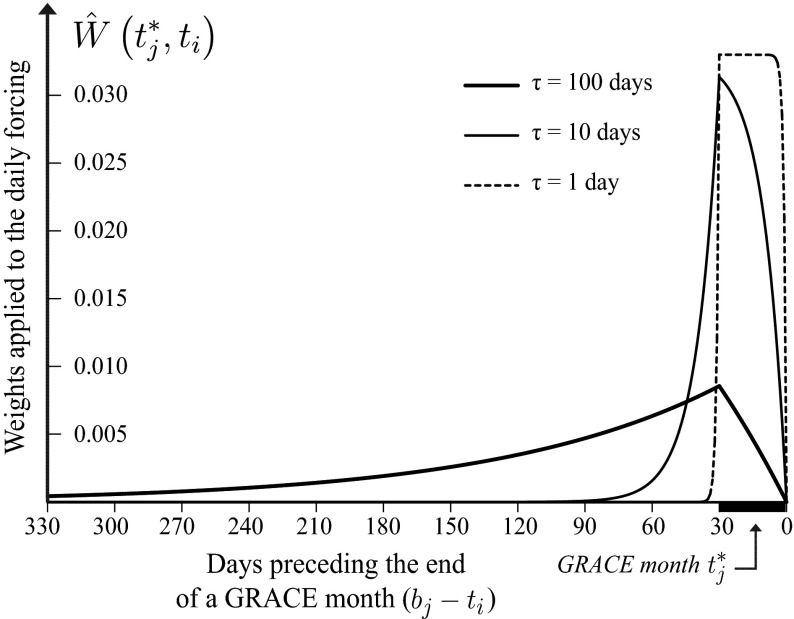
Illustration of the shape of the weighting function (Eq. 9) for different values of the decay time scale *τ*. The *y*-axis corresponds to the normalized weight ($\hat{W}$) that is applied to the daily flux time series when it is averaged to the approximately monthly GRACE resolution

When this is done with a fixed $t_{j}^{*}$ and for all values of $t_{i}$, we obtain the averaging filter illustrated in Fig. 5 for different values of $\tau$—the free parameter controlling the rate of the exponential decay. From this figure, we see that weights are assigned to flux anomalies including to those occurring before the time interval $t_{j}^{*}$. Additionally, we provide a more practical formulation of this weighting function obtained after integrating and normalizing Eq. 6 analytically (Appendix 2):

9 $$\hat{W}\left( {t_{j}^{*} ,t_{i} } \right) = \left\{ {\begin{array}{*{20}l} {\frac{{{\text{e}}^{{\frac{1}{\tau }\left( {t_{i} - a} \right)}} - {\text{e}}^{{\frac{1}{\tau }\left( {t_{i} - b} \right)}} }}{b - a}}&{{\text{if}} \quad a \ge t_{i} } \\ {\frac{{1 - {\text{e}}^{{\frac{1}{\tau }\left( {t_{i} - b} \right)}} }}{b - a}}&{ {\text{if}} \quad a < t_{i} \le b} \\ {0}&{ {\text{if}} \quad b < t_{i} } \\ \end{array} } \right.$$

#### Shape and Properties of the Weighting Function

The parameter *τ* controls the rate of exponential decay and will hereafter be referred to as the *decay time scale* of the weighting function. Inverting Eq. 6 for *τ* shows that *τ* corresponds to the number of time steps (e.g., days) after which the influence of a given flux anomaly $f\left( {t_{i} } \right)$ will have reduced to 1/e ≈ 37 % of its initial influence at time $t_{i}$.

An interesting property is that when *τ* tends to small values (see Fig. 5 for *τ* = 1), $\hat{W}\left( {t_{j}^{*} ,t_{i} } \right)$ converges very quickly to a weighting function that is almost equivalent to the arithmetic mean performed over the interval $\left[ {a_{j} ,b_{j} } \right]$ (i.e. Eq. 3). Small values of *τ* correspond to small decay time scales, indicating that a single flux anomaly will not have a prolonged effect on the state variable. A hydrological interpretation of this feature suggests a short mean residence time of the water store. Inversely, large values of *τ* imply longer residence times and, therefore, more weight is given to anomalies occurring before the time interval of interest. In such a case, it is interesting to note that anomalies occurring near the end of the given time interval are assigned small weights. Hence, the difference between the presented weighting scheme and an arithmetic mean becomes more important for larger *τ*. Since the value of *τ* at each grid cell is unknown in our application, it needs to be estimated from the data. Here, we optimize the agreement between the monthly averaged subseasonal forcing (i.e. *X*_sub_ of $f\left( t \right)$) and the subseasonal monthly GRACE (i.e. *X*_sub_ of $s^{*} \left( {t^{*} } \right)$) by maximizing the squared product–moment correlation coefficient. In the presented study, this weighting function is used for the analysis of the subseasonal component only (Sect. 4.5).

### Significance Testing and Correlation Analysis

#### Linear Trends

A common nonparametric test for detecting monotonic trends in hydro-meteorological time series is the Mann–Kendall rank-based test. However, serial correlation (autocorrelation) in time series has been shown to heavily influence the power of this test (Yue et al. 2002), and several methods have been proposed to address this issue (Hamed and Rao 1998; Yue and Wang 2004; Hamed 2009). Here, we use the modified Mann–Kendall trend test described by Yue and Wang (2004) on deseasonalized GRACE time series (*X*_tot_ − *X*_seas_). In this test, the autocorrelation estimated from the deseasonalized and detrended time series is used to compute an effective sample size, which is then used to correct the Mann–Kendall statistic. In addition, as the trend test is performed locally (i.e. at each grid cell) and due to the high spatial autocorrelation of the GRACE data, there is an increased probability that the null hypothesis is falsely rejected (Wilks 2011). Hence, we additionally control this false discovery rate (FDR) using the approach described by Benjamini and Hochberg (1995), which has shown good performance when applied to climate data (Ventura et al. 2004; Wilks 2006; Gudmundsson and Seneviratne 2015). The trends are considered statistically significant when the *p* value falls below a critical value (*p* < 0.01).

#### Inter-Annual Anomalies

Regarding the inter-annual anomalies (*X*_inter_), the degree of linear association between GRACE and the atmospheric forcing is quantified with the product–moment correlation coefficient. As the inter-annual anomalies correspond to the low-frequency component of the GRACE signal, they exhibit important serial correlation, which prevents the use of conventional hypothesis testing techniques (e.g., *t* test). Here, we use moving block bootstrapping in order to estimate the null distribution of the correlation coefficient at each grid point (Mudelsee 2014). Although there is no standard recommendation on the selection of an optimal block length, a good starting point is to use a block length larger than the decorrelation time (i.e. the number of time steps after which the serial correlation is not significant anymore). Based on this criterion, we find that a block length of 20 months is sufficient for our application. We perform 10,000 bootstrap replications at each grid point and estimate the 95 % confidence intervals from this null distribution. A correlation coefficient is declared significant when it does not belong to the range of the local confidence interval.

#### Seasonal Cycle

Previous studies have shown that there is often a temporal lag between the seasonal cycle of precipitation and terrestrial water storage (e.g., Papa et al. 2008; Ahmed et al. 2011; Frappart et al. 2013). It is also known that water storage and surface temperature are related through evapotranspiration and snow melt; however, differences in the phasing of GRACE versus these atmospheric variables were, to our knowledge, never surveyed at a global scale. We define the phase shift as the lag (in months) minimizing the residual sum of squares between the standardized seasonal cycles of both GRACE and the atmospheric forcing. When these paired seasonal cycles strongly differ in shape, this procedure can sometimes lead to meaningless lag values. A  t-test of the Pearson product–moment correlation between the time–lagged seasonal cycles is used to filter out these potentially misleading values (*p* < 0.01). We additionally control the FDR following Benjamini and Hochberg (1995).

#### Subseasonal Residuals

Similarly as for the inter-annual anomalies, the product–moment correlation coefficient is used to quantify the degree of linear association between GRACE and the atmospheric forcing data. The subseasonal residuals (*X*_sub_) correspond to the high-frequency component and are thus the least affected by serial correlation. However, we found that these time series still contain minor but significant serial correlation (not shown). For consistency, we thus use an identical significance testing setting as for the inter-annual anomalies (i.e. a moving block bootstrapping).

### Identifying Droughts in the GRACE Record

Here we investigate the *average storage deficit* during drought events identified using an approach based on Thomas et al. (2014). This approach defines 1) storage deficit as a negative departure (in mm) from the seasonal cycle and 2) drought duration as the number of months with continuous deficits. The *average storage deficit* simply corresponds to the arithmetic mean of the storage deficit observed during a given drought event and is used as a measure of average drought intensity. Here two differences compared to Thomas et al. (2014) are introduced.

First, we remove the linear trends from the time series prior to drought identification. The reason is that strong linear trends can result in one end of the time series being systematically above/below the seasonal cycle. In such a case, the proposed method would have a tendency to underestimate/overestimate the magnitude of dry events. Hence for the purpose of this study, linear trends are removed prior to the analysis and potential decadal drying trends are discussed in a separate section. Our analysis is thus based on the sum of the inter-annual and subseasonal components only (*X*_inter_ + *X*_sub_, also see Fig. 2). Occasionally, drought events occurring at the end or the beginning of the time series can be large enough to influence the trend estimate itself, even when using the Theil–Senn slope to reduce this effect. Hence, it is important to note that, in some cases, removing the linear trends may cause an underestimation of the drought intensity.

Second, the minimum duration for considering a drought event is defined as a period of three consecutive months with water storage deficit. Unlike Thomas et al. (2014), we apply this criterion only to the inter-annual component *X*_inter_ (see Fig. 2) and not to the sum of the inter-annual and subseasonal components (*X*_inter_ + *X*_sub_). The reason is that, compared to the basin-scale assessment of Thomas et al. (2014), subseasonal variability (*X*_sub_) is larger at the grid level and including it would otherwise considerably reduce the probability of observing long periods with consecutive deficits.

## Global Hydrological Variability in the GRACE Data

### Distribution of GRACE Variance Among Temporal Components

**Fig. 6:**
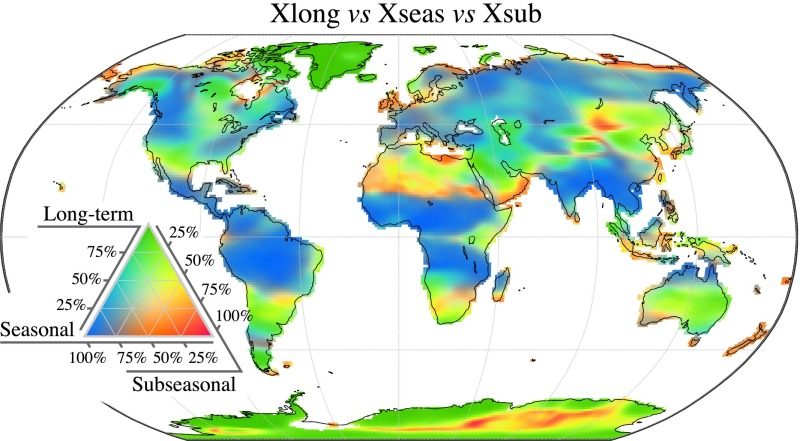
Distribution of the total GRACE variance among the long-term (*green*), seasonal (*blue*) and subseasonal (*red*) components, expressed in per cent of the total variance, indicating the dominant modes of temporal variability in terrestrial water storage for different regions

The relative magnitude of the three components extracted from the STL procedure (*X*_long_, *X*_seas_ and *X*_sub_) can be evaluated by comparing each component’s variance to that of the total signal. As shown in Fig. 6, the relative magnitude of each of the different components is subject to high spatial variability across the world. To ease the interpretation, Fig. 6 can also be compared to the standard deviation of the total signal in Fig. 1. As already identified in early studies (Wahr et al. 2004), the seasonal cycle is dominant in many tropical regions like the Amazon basin, Central Africa and India. A notable exception is the Indo-Australian archipelago where the GRACE signal is heavily perturbed by signal leakage from the ocean as well as gravity anomalies consecutive to the 2004 Sumatra earthquake. The seasonal cycle is also dominant at higher latitudes, particularly in Siberia and in north-western America, although these regions do not have the largest variance in absolute terms.

Subseasonal variability (*X*_sub_) is dominant in regions where the GRACE signal has already a relatively low variance (Fig. 1) and is most likely dominated by noise such as in the Sahara desert. Although we do not further investigate this matter, it is interesting to note that Arctic coastal regions such as the coasts of Northeast Siberia and Canada seem to be mostly affected by subseasonal variability.

We also observe that many regions of the world are dominated by inter-annual variability (*X*_long_). The signal found in Greenland and Antarctica, parts of Alaska and the Hudson Bay is the result of the interplay between ice mass loss, other water storage changes and glacial isostatic adjustment. As a result, these regions require a specific treatment before conclusions can be drawn concerning the dominant features of hydrological variability (Velicogna et al. 2014). Other regions particularly dominated by long-term variability include the south-western Central USA as well as the Middle East, some of which are already documented in the literature as being influenced by decadal droughts and long-term trends in groundwater storage (Long et al. 2013; Voss et al. 2013; Forootan et al. 2014b). Other interesting features include the Lake Victoria and the Aral Sea where long-term surface water variations can be related to both human activities and climate variability (Swenson and Wahr 2009; Singh et al. 2012). Finally, some regions in the southern hemisphere like Australia and Argentina were also shown to exhibit an important inter-annual variability that can be related to the El-Niño Southern Oscillation (ENSO) (García-García et al. 2011; Abelen et al. 2015).

### Linear Trends

In this section, we will assess in further detail the relative importance of linear trends (*X*_lin_) versus nonlinear inter-annual variability (*X*_inter_) by looking at the magnitude of each of these two components in the long-term variability (*X*_long_):

10 $$R_{{{\text{lin}}/{\text{long}}}} = \frac{{\sigma_{\text{lin}}^{2} }}{{\sigma_{\text{long}}^{2} }} = 1 - \frac{{\sigma_{\text{inter}}^{2} }}{{\sigma_{\text{long}}^{2} }}$$

**Fig. 7:**
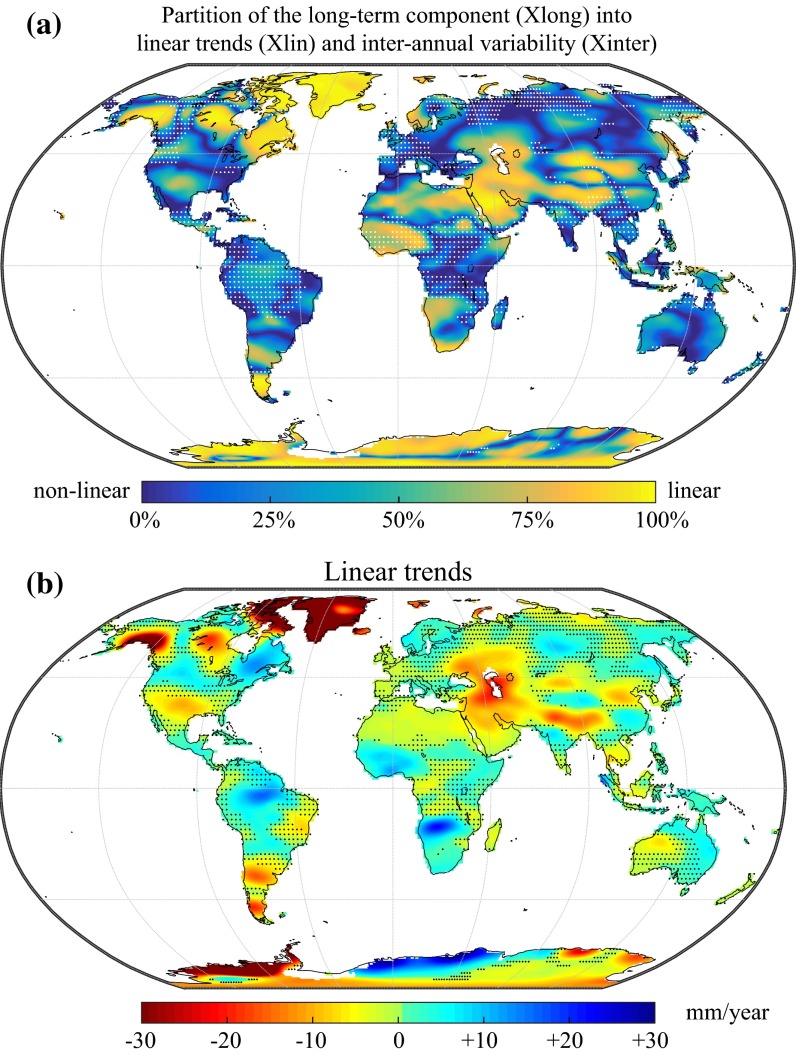
**a** Fraction of the long-term variability that corresponds to a linear trend, expressed in per cent of the long-term variance. Large values (*yellow*) indicate that most of the long-term variability corresponds to a linear trend. Small values (*blue*) indicate that (non-linear) inter-annual variability is dominating. Stippling indicates regions where the long-term variability represents less than 20 % of the total GRACE variance (see also Fig. 6). **b** Magnitude of linear trends in the GRACE signal (expressed in mm/year). Stippling indicates regions with non-significant trends (*p* < 0.01). Note that the colour scale is truncated for negative trends beyond −30 mm/year

This formulation is also equivalent to the coefficient of determination of the linear trend as estimated by the Theil–Sen slope with respect to the long-term component. The colour scale in Fig. 7a represents the ratio of the linear trend variance to that of the whole long-term component (Eq. 10) and shows how the long-term component (*X*_long_) variance is partitioned between linear (*X*_lin_) and nonlinear trends (*X*_inter_). The sign, magnitude and significance (*p* < 0.01) of the linear trends are shown in Fig. 7b. Note the truncated colour scale for negative trends beyond −30 mm/year.

We observe that the long-term variability in large areas of Greenland and Antarctica is dominated by a linear trend. This can be related to ice mass loss once glacial isostatic adjustment has been accounted for (Ramillien et al. 2006; Velicogna and Wahr 2006; Chen et al. 2006b; Wouters et al. 2008; Velicogna 2009; Velicogna and Wahr 2013). Examples of other regions of the cryosphere concerned with documented linear trends include Alaskan mountain glacier melting (Chen et al. 2006a; Arendt et al. 2013; Larsen et al. 2015) and icefield melting in Patagonia (Chen et al. 2007b; Ivins et al. 2011). Strong linear trends located close to the Hudson Bay have been related to glacial isostatic adjustment (Tamisiea et al. 2007), and recent studies focusing on Arctic regions showed that both isostatic and hydrological trends contribute to the observed signals (Frappart et al. 2011a; Wang et al. 2013).

Many pronounced negative trends can also be observed in other regions of the world. One of the most recognized drying trends occurs in north-west India and is related to groundwater depletion (Rodell et al. 2009; Chen et al. 2014). Most of the long-term signal that is dominating GRACE variability over the Middle East, the Caspian Sea and the Aral Sea can be attributed to a drying trend partly due to anthropogenic water abstraction (Singh et al. 2012; Voss et al. 2013; Joodaki et al. 2014; Forootan et al. 2014a). On the contrary, the region of Lake Victoria is dominated by nonlinear variations in the long-term component, which have been related to hydropower dam operations, precipitation anomalies and ENSO (Awange et al. 2008; Swenson and Wahr 2009; Becker et al. 2010; Hassan and Jin 2014). Another important drying trend can be found in Argentina, especially in the southern part of the La Plata basin (Chen et al. 2010b; Abelen et al. 2015). Finally, the drying trend documented by Crowley et al. (2006) in the Congo river basin for the period 2002–2006 is found to be insignificant based on the current analysis.

The significance analysis also identified regions with trends which have not been identified yet or have only drawn little attention so far. For instance, the extended drying trends located to the North of both the Black Sea and the Caspian Sea could be potentially investigated in more detail. Interestingly, small but statistically significant drying trends (−3 mm/year) can also be found over large portions of the Sahara desert. So far, little attention has been devoted to GRACE variability in this region as most of the signal is contaminated by noise. Nevertheless, these drying trends have been partly documented (Ahmed et al. 2014; Ramillien et al. 2014) and to some extent attributed to groundwater extraction from fossil aquifers in the Sahara region.

Significant positive trends can also be found in southern Africa, near the Upper Zambezi and Okavango river basins as well as in the Sahel and the Niger basin, and these trends have already been well documented (Ramillien et al. 2014). In a comparison with rainfall observations from different sources, Ahmed et al. (2014) have found that the increasing trend in the Niger basin could be related to an increase in precipitation; however, this was not the case for the Upper Zambezi and the Okavango basins. Although Ahmed et al. (2014) suggest that this could be due to longer residence times in these watersheds, we feel that more investigation is still required to explain the very strong positive trend in this region. A positive trend is also found in the Amazon basin and has been described, for instance, in Chen et al. (2010a) and could, to a certain extent, be related to precipitation anomalies based on an analysis of the period 2003–2008 by Xavier et al. (2010).

The linear trends derived over north-western China raise some concerns with respect to a possibly spurious origin. The alternating bipolar patterns of positive and negative trends, oriented along the meridian 100°E, could be due to some accidental disturbance originating in the processing of the GRACE data. A very similar pattern could already be found in Fig. 8 of Frappart et al. (2011b), which compared trends derived after different postprocessing methods for the period 2003–2008. It is possible that these trends found over China are specific to the destriping algorithm of Swenson and Wahr (2006) since they are not reproduced by the other postprocessing methods investigated by Frappart et al. (2011b). However, Feng et al. (2013) were also able to relate drying trends in the Beijing region to groundwater observations. Consequently, special care should be taken when interpreting trends from the current GRACE Tellus estimates in that region.

### Inter-Annual Anomalies

**Fig. 8:**
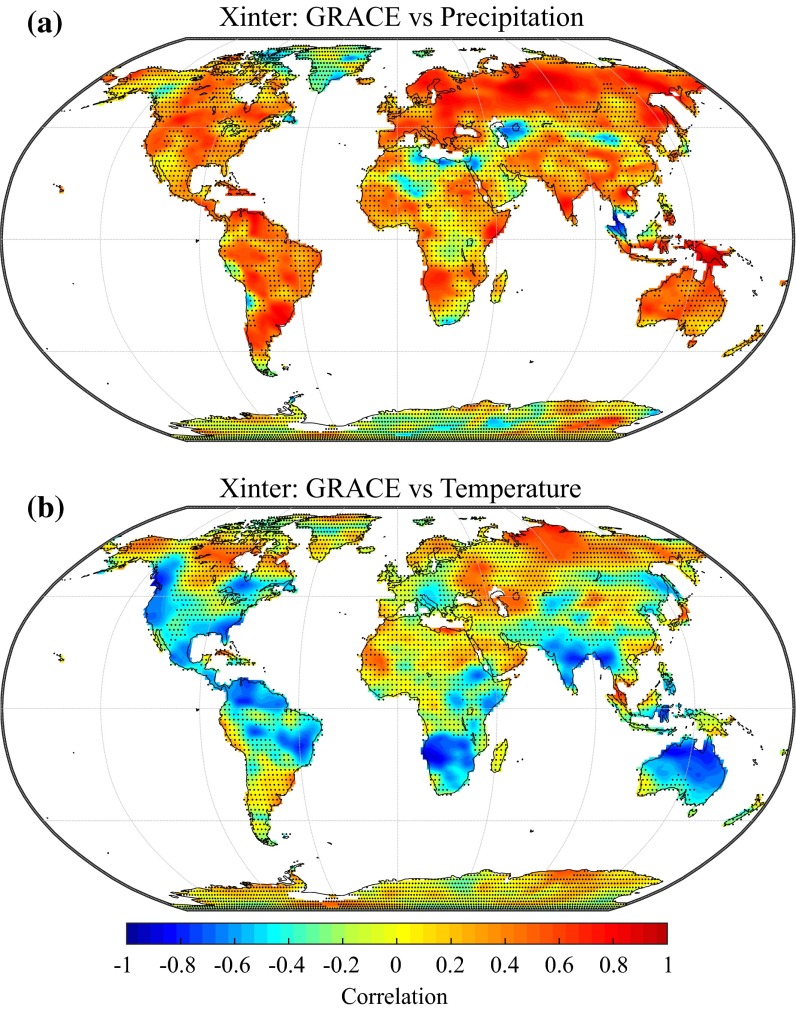
Correlation between the inter-annual variability of water storage and **a** precipitation, **b** temperature. Stippling indicates regions with non-significant correlation coefficients (*p* < 0.05)

The inter-annual anomalies correspond to the nonlinear part of the long-term component (Eq. 1). In this section, we assess the degree to which the inter-annual anomalies derived from the GRACE time series can be correlated with the inter-annual anomalies of the atmospheric forcing. Figure 8a depicts the correlation between the inter-annual water storage and precipitation anomalies. We observe moderately high positive correlations (>0.6) between these two components for parts of Europe, Russia and North America, which indicate a positive effect of precipitation on terrestrial water storage. Correlations are more erratic over subtropical and equatorial regions, possibly resulting from deficiencies in the ERA-Interim precipitation forcing, which are known to be more pronounced, for instance, over Africa and South America (Simmons et al. 2010). In these regions, other precipitation datasets based either on ground measurements or satellite observations may give different results. For example, we find relatively low correlations between inter-annual water storage and precipitation over the region of the African Great Lakes; however, Becker et al. (2010) found a seemingly good agreement with GRACE when using monthly precipitation data from the Global Precipitation Climatology Project (GPCP). For Africa and South America, Morishita and Heki (2008) found that mass changes from GRACE could be related to precipitation anomaly patterns extracted from the Climate Prediction Center Merged Analysis of Precipitation (CMAP). Over the Amazon, Chen et al. (2010a) related inter-annual water storage change to precipitation anomalies (from GPCP) and ENSO indices, while Frappart et al. (2013) found that the inter-annual variability of water storage was in reasonable agreement with precipitation from the Tropical Rainfall Measuring Mission (TRMM). Although correlation coefficients are mostly positive, significant negative correlations can occasionally be found but seem either accidentally caused by perturbations of the long-term gravimetric signal by large earthquakes (e.g., Sumatra region) or would need to be confirmed in a regional investigation (Caspian Sea area).

The same analysis was performed with ERA-Interim 2 m temperature (Fig. 8b). We find that the correlation between inter-annual water storage and temperature is negative in most cases. Correlations are moderately strong over parts of North America, South America, southern Africa, India and Australia. Such a negative relationship is usually expected not only since temperature is an important driver for evaporative demand but also because low moisture availability can result in higher temperatures (Seneviratne et al. 2010; Mueller and Seneviratne 2012). However, correlations found in the southern hemisphere could also be related to confounding factors such as atmospheric circulation patterns (e.g., ENSO) and hence do not necessarily indicate a direct link between temperature and terrestrial water storage. In addition, significant positive correlations between long-term temperature and water storage anomalies can be found over parts of Siberia. Long-term water storage anomalies in this region may be related to interactions with permafrost although such relationships are still unclear at this stage (Velicogna et al. 2012; Vey et al. 2013).

Non-significant correlations can be due either to other unidentified sources of long-term variability in the GRACE data or errors and biases in the long-term variability of the ERA-Interim atmospheric forcing. However, the absence of correlation with either precipitation or temperature in some regions could also indicate that long-term variability in the atmospheric forcing is not controlling or controlled by terrestrial water storage, i.e. that there is no obvious relationship between these variables at the inter-annual time scale. In addition, the literature covered in the section on linear trends already showed that anthropogenic groundwater withdrawal and dam operations could be responsible for long-term changes in the terrestrial water storage variations retrieved from GRACE. Finally, we note that a very large number of locations exhibit moderate correlations (between 0.3 and 0.5 in absolute value), which are actually not significant once serial correlation is taken into account in hypothesis testing (using bootstrapping). This is also an indication that analyses of the inter-annual variability of water storage would greatly benefit from the longer record length that may be provided in the future by the GRACE Follow-On mission scheduled for launch in 2017.

### Seasonal Cycle

**Fig. 9:**
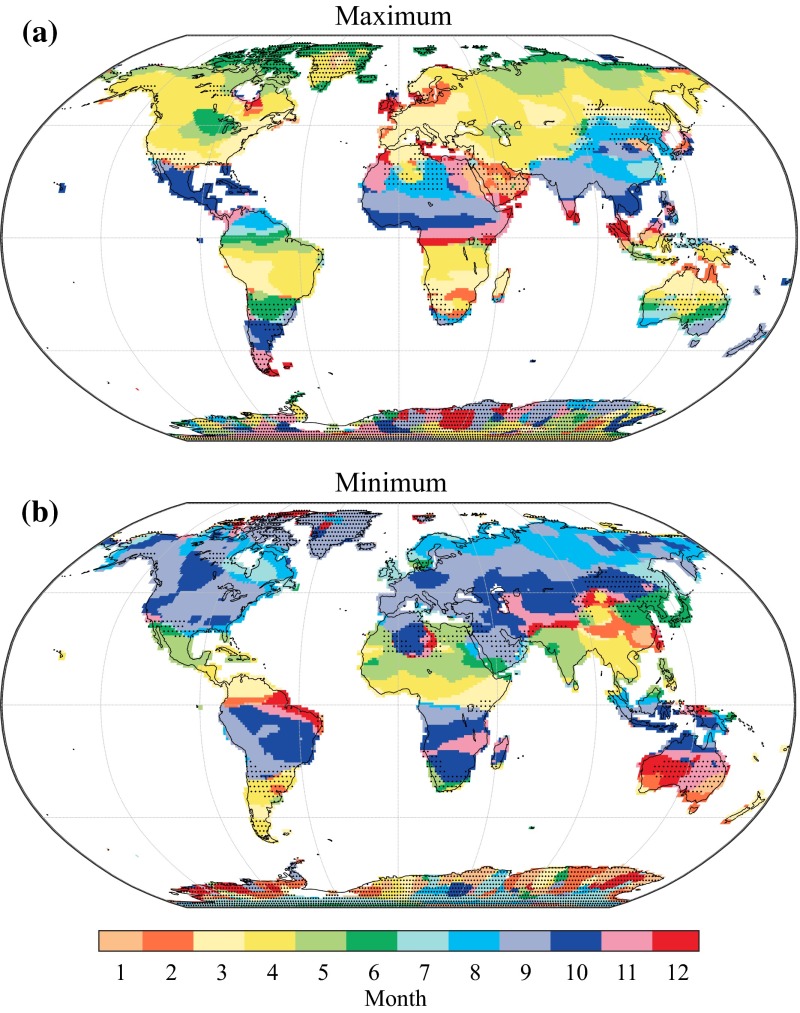
**a** Month with the maximum of the seasonal cycle of water storage. **b** Month with minimum seasonal water storage. Stippling indicates regions where the seasonal variability represents <20 % of the total GRACE variance

The STL decomposition provides a data-driven way of estimating the seasonal cycle which, in contrast to the common practice, does not rely on harmonic models (fitted sines and cosines, e.g., Wahr et al. 2004; Hinderer et al. 2006; Schmidt et al. 2008b). Here we characterize GRACE seasonality by mapping the months with the *maximum* and the *minimum* of the seasonal cycle of water storage and show that they generally follow latitudinal bands (Fig. 9a, b). In the Northern Hemisphere, the peak in terrestrial water storage generally occurs in spring for the cold and temperate regions and in autumn for the subtropical regions (and vice versa in the Southern Hemisphere). The minimum water storage occurs in autumn for the cold and temperate regions and in spring for the subtropical regions (and oppositely in the Southern Hemisphere). In subarctic regions, there is a clear latitudinal trend towards a later maximum, likely corresponding to the delayed response of snow melt to temperature at higher latitudes. Interestingly, the seasonal maximum appears to be also delayed near large inland reservoirs (e.g., the Great Lakes and the Caspian Sea), which potentially reflects the influence of run-off and storage processes and could be subject to further investigations. In most regions, the months with maximum and minimum terrestrial water storage are spaced by an interval of 6 ± 1 months. However, this is not always the case: in northern India, the maximum terrestrial water storage occurs in September and the minimum in May, which is consistent with the effect of the June–September monsoon. These maps can, for instance, be directly compared with hydrological models (see Fig. 6 in Güntner et al. 2007b for an example with a closely resembling colour scale). In addition, it is worth mentioning that the seasonal cycle of water storage over African regions located close to the equator (e.g., the Congo basin, Lake Victoria) exhibits a strong secondary peak, which likely corresponds to the oscillation of the inter-tropical convergence zone (not shown). This secondary peak is also present—although less pronounced—over Ecuador, southern India and Indonesia but is completely absent over the Amazon basin (not shown).

**Fig. 10:**
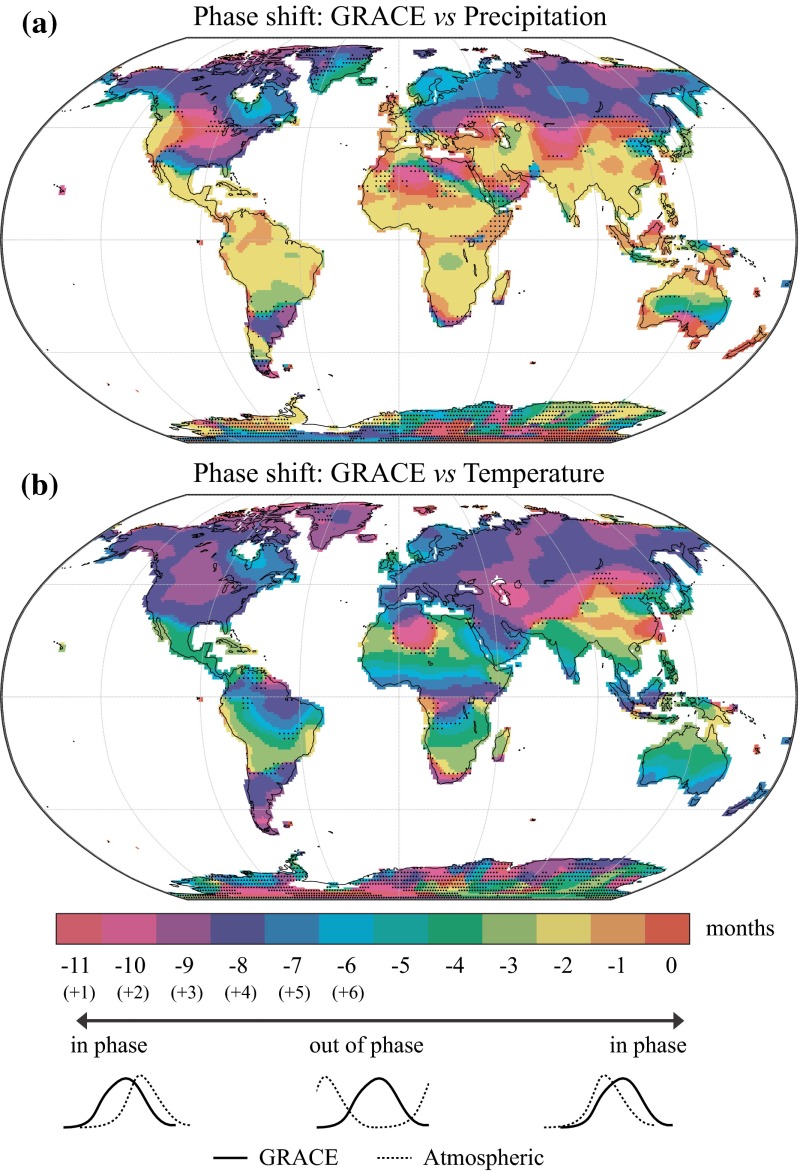
Phase shift (in months) between the seasonal cycle of water storage and the seasonal cycle of **a** precipitation and **b** temperature. Small phase shifts (−1, 0 or −11(+1) months) indicate that the atmospheric forcing is nearly in phase with water storage, whereas large phase shifts (−7, −6 or −5 months) indicate that they are out of phase. Stippling indicates regions where the correlation between optimally phase shifted seasonal cycles is not significant (*p* < 0.05)

The phase shift between GRACE and the seasonal cycles of precipitation and temperature is shown in Fig. 10a, b. Over most tropical and subtropical regions, the seasonal peak of precipitation typically occurs 1–2 months *earlier* than the peak in water storage, likely due to the effect of storage processes. Very similar lags have been found for the Amazon subbasins (see Table 3 in Frappart et al. 2013), for selected regions over central Africa (see Fig. 3 in Ahmed et al. 2011) as well as by Rieser et al. (2010) over Australia (all three studies used satellite precipitation data from TRMM). On the contrary, subarctic and inland temperate regions experience the highest precipitation during the warmer summer months, approximately 3–5 months *later* than the spring maximum in water storage. For coastal subarctic areas, the precipitation maximum tends to occur in autumn due to greater temperature differences between the ocean and land, resulting in a 5- to 7-month phase shift between water storage and precipitation (e.g., Alaska, British Columbia and Scandinavia). More details concerning the phasing of GRACE with snow storage and discharge measurements can be found in Frappart et al. (2011a).

The seasonal cycle of temperature is generally out of phase with respect to the seasonal cycle of water storage (Fig. 10b). In most temperate and subarctic regions, the peak temperature typically occurs in summer, 2–3 months earlier than the autumn minimum in water storage. Over tropical regions, the seasonal cycle of temperature is completely opposed to the water storage cycle, with corresponding phase shifts of 4–7 months. This anti-phasing between water storage and temperature is likely related to the effects of both temperature and radiation on evapotranspiration. Over equatorial regions, the seasonality of temperature is much less pronounced but still lagging the water storage cycle by 3–4 months. The southern part of China exhibits a very specific pattern, with maximum temperatures occurring in summer and seasonal water storage peaking in late summer, resulting in an almost perfect phasing between water storage and temperature.

### Subseasonal Residuals

**Fig. 11:**
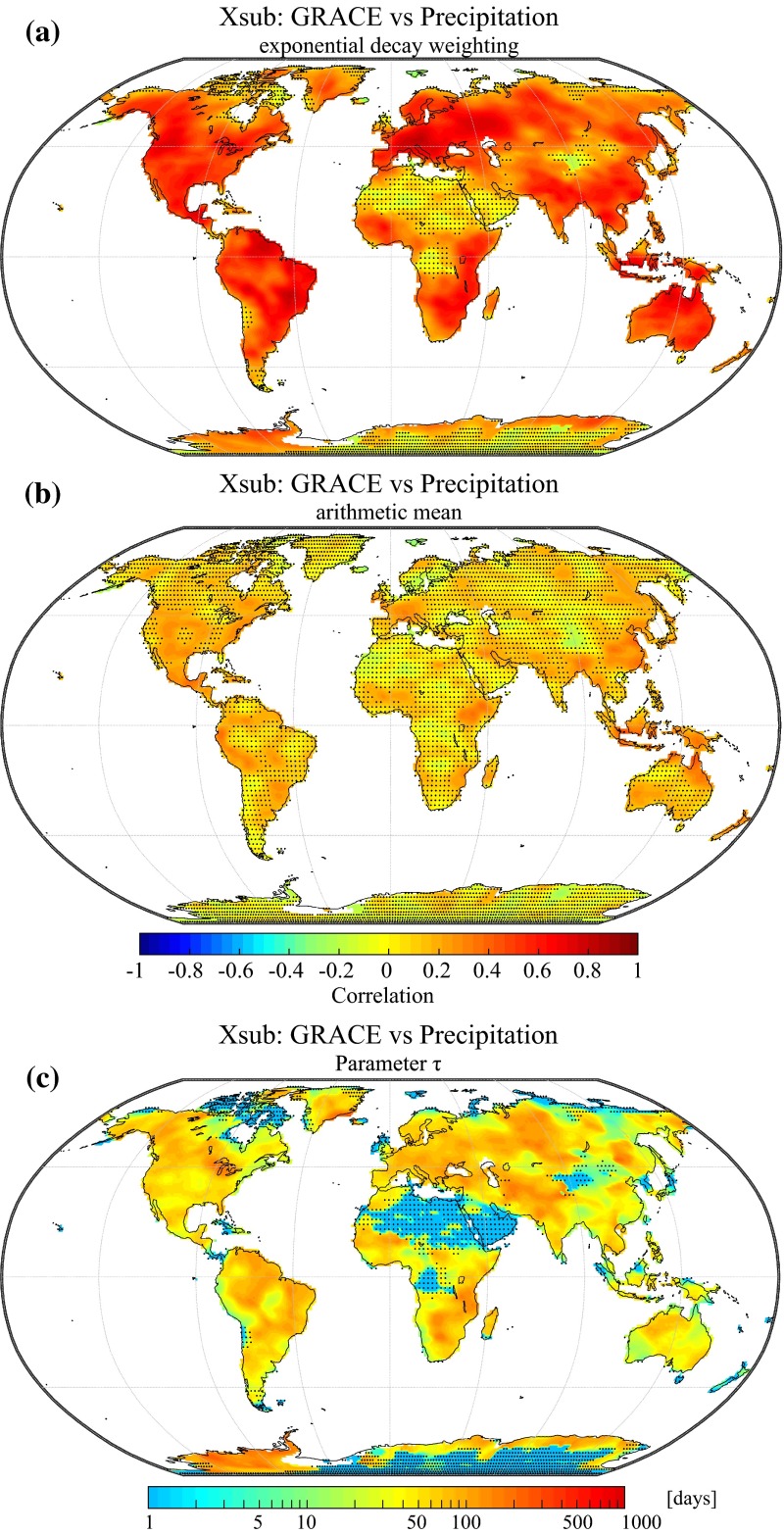
**a** Correlation between the subseasonal variability of water storage and precipitation averaged with the weighting function introduced in Sect. 3.2 (Eq. 9). **b** Correlation between the subseasonal variability of water storage and precipitation averaged using the arithmetic mean (Eq. 3). **c** Value of the calibrated decay parameter (*τ*) with units of days. Stippling indicates regions with non-significant correlation coefficients (*p* < 0.05)

Figure 11a shows the correlation between the high-frequency components of GRACE and precipitation averaged with the new averaging scheme presented in Sect. 3.2. Significant positive correlations are found over many regions of the world, indicating that a large fraction of high-frequency GRACE variability can be statistically related to short-term anomalies of the precipitation forcing. Interestingly, significant correlations can also be found over large portions of Indonesia, although the GRACE signal in this region is usually believed to be strongly deteriorated by signal leakage from the ocean. A possible explanation might be that short-term precipitation variability in this tropical monsoon region is large enough to overcome the higher errors associated with coastal and insular regions. A notable exception to the global pattern is the Congo river basin where no significant correlations can be found. This area corresponds to a major convective region for the global climate system which is still poorly represented by atmospheric reanalyses in comparison with other regions (Washington et al. 2013). Many extremely arid regions also show non-significant correlations (Sahara, Atacama, Taklamakan and Gobi deserts), confirming the view that high-frequency GRACE variability in these regions is dominated by noise.

**Fig. 12:**
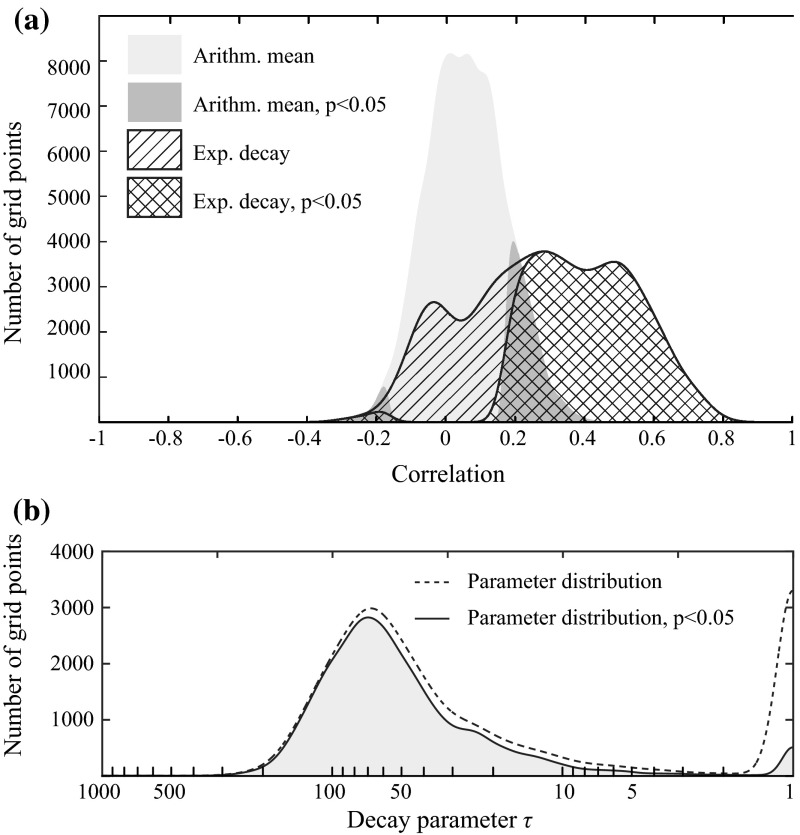
**a** Distribution of the correlation coefficients obtained with a simple average of the precipitation forcing (*grey surface*) versus the distribution of the correlation coefficients obtained with the newly introduced weighting function (*hatched surface*). The part of significant correlation coefficients (*p* < 0.05) is indicated using a darker colour or double hatching. **b** Distribution of the calibrated decay parameters used in the weighting function. Note the base 10 logarithmic scale of the *x*-axis

In order to assess the influence of the new averaging method, we can visually compare the correlations shown in Fig. 11a to the correlations obtained with a simple monthly arithmetic mean of the daily residuals (Fig. 11b). The exponential decay approach enhances the correlations over most regions of the world, with an average increase of +0.3 (excluding regions which exhibit non-significant correlations in Fig. 11a). Figure 12a enables an even more direct comparison between the distributions of the correlations shown in Fig. 11a, b. This illustrates the value of the proposed weighting scheme and reveals that using monthly arithmetic averages of precipitation may have resulted in underestimating the relation with water storage on the subseasonal time scale. This finding is of particular interest for studies comparing GRACE data to monthly precipitation time series (e.g., Forootan et al. 2014a) which typically make use of monthly precipitation averages.

Values of the calibrated decay parameter *τ* used to compute the monthly averages from the daily precipitation data are shown in Fig. 11c. Overall, decay time scales exhibit systematic spatial variability that could potentially be related to many different factors, including climatic conditions as well as soil and vegetation characteristics. The probability density distribution of this parameter is also shown in Fig. 12b, and we find that significant values generally range between 10 and 200 days with a median value of approximately 50 days. Based on the weighting function (Eq. 9), it can be calculated that for the median value of *τ* = 50 days, the precipitation residuals of the first 100 days preceding the beginning of a GRACE month account for 65 % of the monthly average. On the contrary, days covered by the time interval of a given GRACE month account for only 25 % of the monthly average. This shows that, on the subseasonal time scale, precipitation preceding a GRACE month usually has a higher impact on correlations with terrestrial water storage than the precipitation of the coinciding month. This is due to the fact that the influence of high-frequency precipitation anomalies on regional hydrology tends to decay with time so that precipitation events occurring just before or at the very beginning of a GRACE month have a higher impact on the average water storage of a given month. Conversely, precipitation anomalies occurring during or at the very end of a GRACE month have a lower impact on the water storage anomalies, or may even occur after the latest GRACE overpass.

**Fig. 13:**
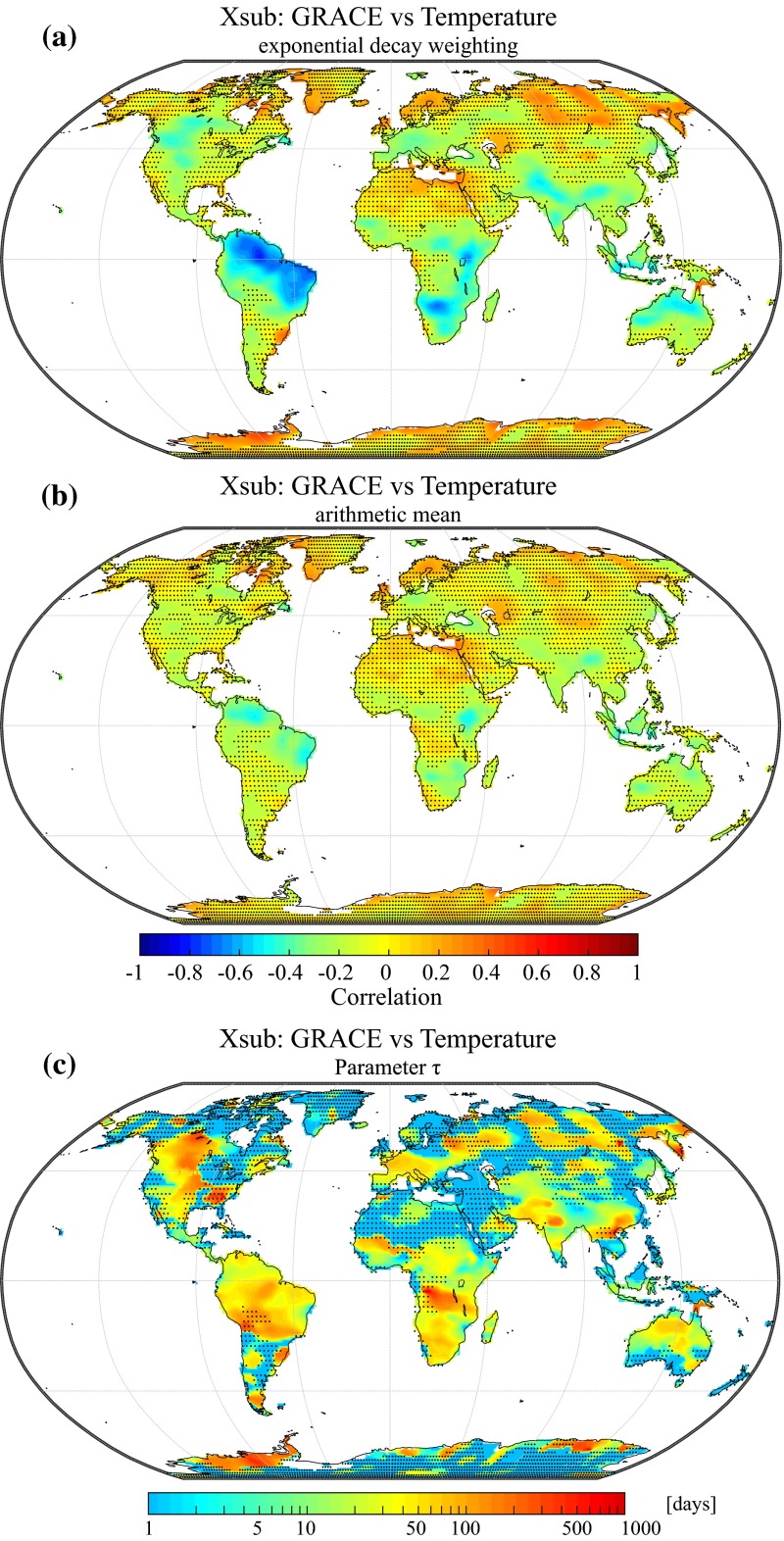
**a** Correlation between the subseasonal variability of water storage and temperature averaged with the weighting function introduced in Sect. 3.2 (Eq. 9). **b** Correlation between the subseasonal variability of water storage and temperature averaged using the arithmetic mean (Eq. 3). **c** Value of the calibrated decay parameter (*τ*) with units of days. Stippling indicates regions with non-significant correlation coefficients (*p* < 0.05)

Figure 13a shows the results of the same analysis performed with the temperature data. It reveals regions where the integrated effect of antecedent temperatures can be statistically related to water storage anomalies. Temperature is one of the main controls for evaporative demand, and hence, negative correlations are expected and can indeed be found over many regions of the world, especially over South America, South Africa, the region of the African Great Lakes, India, Indonesia and northern Australia. As for precipitation, the use of the exponential decay approach leads to enhanced correlations when compared to the arithmetic mean (Fig. 13b). However, improvements are less important than for precipitation and are often concentrated in regions where a significant relationship can already be found with the simple mean. Decay time scales over these regions (Fig. 13c) generally fall between 1 to 3 months, yielding a data-driven first-order estimate of how long temperature anomalies can significantly impact the subsequent state of terrestrial water storage. Positive and significant correlations can occasionally be found over some areas, notably over Siberia, Scandinavia and Antarctica. For these regions, we hypothesize that these positive correlations could reflect the tendency of warm winters to be more humid in comparison with cold winters. On the other hand, warm summers are also expected to increase snow melt so that we cannot come to a definitive conclusion for these regions based on the presented results.

### Droughts

**Fig. 14:**
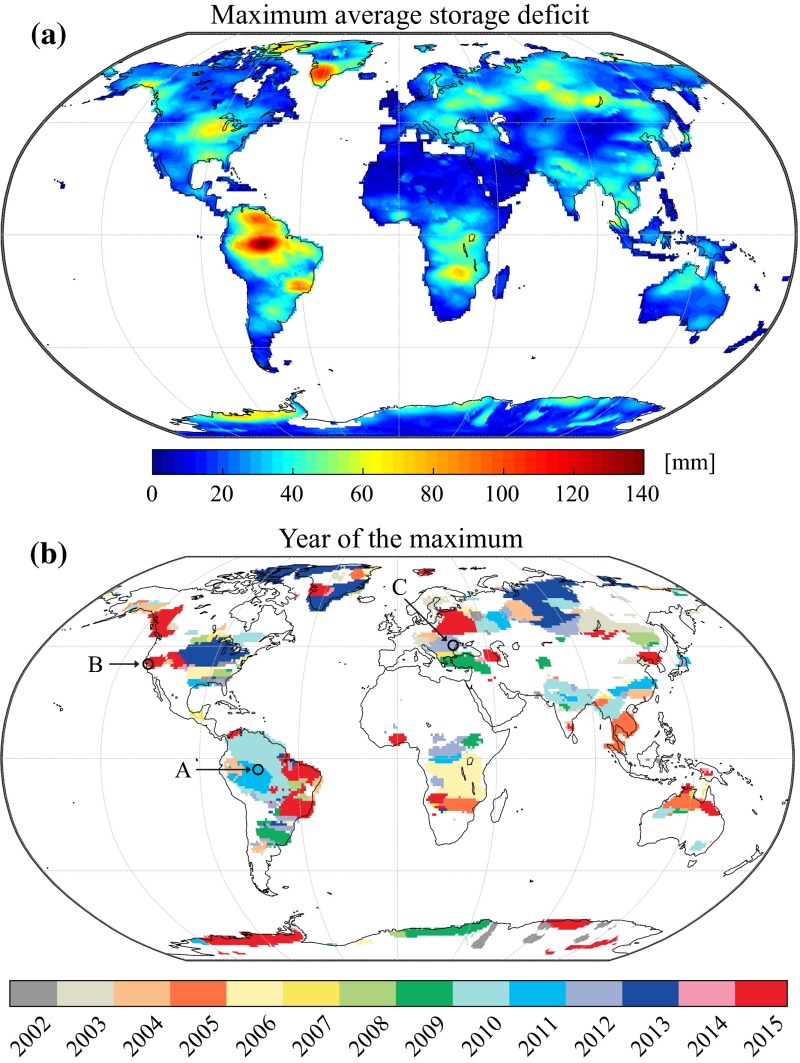
**a** Maximum value of the average storage deficit observed in the period April 2002–August 2015, expressed in mm of equivalent water height. **b** Year corresponding to the maximum value of the average storage deficit, showing only regions with a deficit larger than 30 mm. Letters *A–C* correspond to the location of the time series in Fig. 15

**Fig. 15:**
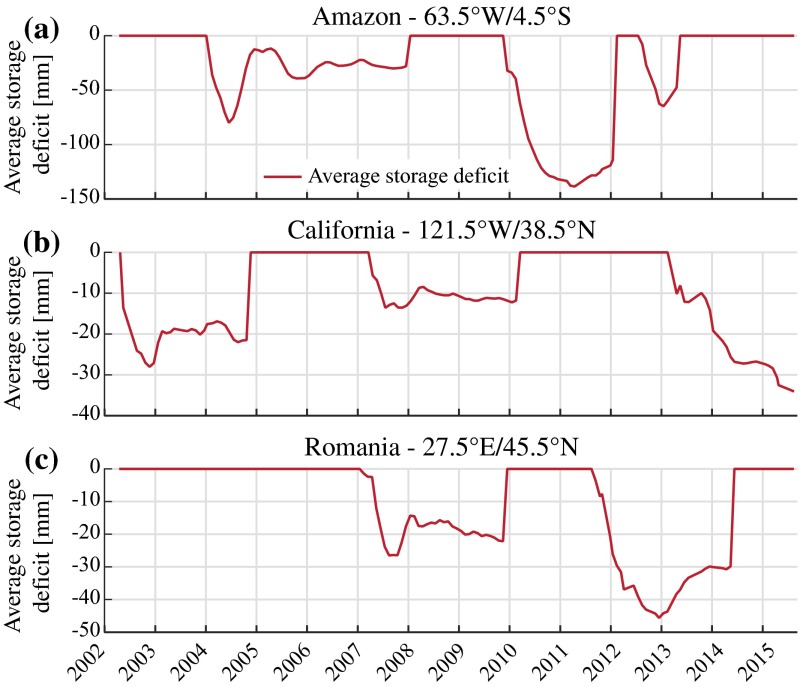
Time series of average water storage deficit for grid cells (see location in Fig. 14b) in **a** Amazon (63.5°W/4.5°S), **b** California (121.5°W/38.5°N), **c** Romania (27.5°E/45.5°N)

In Fig. 14a, we show the maximum *average storage deficit* (see Sect. 3.4) ever observed for all drought events identified in the GRACE record. The year corresponding to this maximum is depicted in Fig. 14b for events with a magnitude larger than 30 mm. This threshold was chosen to mask out smaller features which are difficult to interpret in a global assessment but may still be relevant in a regional context. Many droughts that have previously been documented in the GRACE literature can be identified, notably the 2010 Amazon drought, which is additionally illustrated in Fig. 15a. Drought events in the Amazon basin were shown to be related to precipitation deficits and ENSO (Davidson et al. 2012; Frappart et al. 2013). A multi-year drought is also found for the period 2004–2008 (Fig. 15a) and likely corresponds to the multiple consecutive dry years identified, for instance, by Frappart et al. (2012) and Thomas et al. (2014). Note that this is related to the chosen drought metric, which might not capture all relevant aspects. The ongoing drought in the Central Valley of California is also identified in Fig. 14b, and the time series of the average storage deficit (Fig. 15b) shows that this region also suffered from multiple dry episodes in previous years. This was already identified in previous studies which related these recurrent drought events to severe groundwater depletion (Famiglietti et al. 2011; Famiglietti 2014; Chen et al. 2015). Other documented events identified in Fig. 14b include the 2008–2009 drought in the La Plata basin (Abelen et al. 2015), the 2010–2013 drought in Texas (Long et al. 2013), the 2007–2009 drought in the south-eastern USA (Houborg et al. 2012) and the 2012–2015 North American drought (Chew and Small 2014; Hoerling et al. 2013). We also identify the 2006–2007 dry conditions over Lake Victoria (Swenson and Wahr 2009) and the African Great lakes (Becker et al. 2010) and the 2006–2008 drought in the Zambezi basin (Thomas et al. 2014), which are, in this analysis, captured together as a large-scale and spatially contiguous event. Drought conditions can also be found in northern India for the period 2009–2010 even though the linear trend due to groundwater depletion has been removed from the data prior to drought identification. The year 2009 was indeed shown to be the driest year of the decade for this region in terms of precipitation (Chen et al. 2014) and resulted in higher groundwater abstraction rates. Our analysis shows that the average storage deficit was consecutively maximal in 2010. The Sumatra region also exhibits an important “deficit” which, as confirmed by a local investigation, is probably an artefact caused by the 2004 earthquake. In Australia, multi-year droughts have been related to precipitation deficits (García-García et al. 2011). However, due to the long duration of these decadal drought events, the average storage deficit is lower. Our results also reveal undocumented features found in the GRACE record, such as a dry event from 2012 to 2014 over south-eastern Europe (Figs. 14b, 15c) as well as a severe drought in the Sao Paulo region and a moderate drought over North Korea in 2015 (both still ongoing at the time of writing). A dry period can also be identified during 2010–2011 to the North of the Caspian Sea and is likely associated with the 2010 Russian heatwave. Many other events can also be found over central Russia and were, to our knowledge, never identified using GRACE data.

## Conclusions

In this study, we have decomposed the GRACE time series into (1) linear trends, (2) nonlinear inter-annual anomalies, (3) seasonal cycles and (4) subseasonal residuals. The relative importance of each of these components with respect to the original GRACE signal has been evaluated, allowing for a global assessment of the dominant features of temporal variability in terrestrial water storage. In most cases, the GRACE signal is dominated by seasonal or/and long-term variability, while subseasonal variability generally accounts for a small fraction of the total signal variance. Partitioning the long-term variability into linear trends and nonlinear components reveals that some regions are dominated by linear trends, while nonlinear inter-annual variability is prevalent in others. The magnitudes of the linear trends have been quantified using the robust Theil–Sen estimator, reproducing many already documented trends but also revealing some features that had not been identified previously. In addition, the significance of the trends was evaluated using statistical hypothesis testing techniques which take serial correlation (autocorrelation) into account, contrasting the common practice in the GRACE literature.

In a more detailed analysis, each component of temporal variability (except linear trends) has been compared with equivalent components extracted from daily precipitation and temperature time series of the atmospheric reanalysis ERA-Interim:

- *Inter*-*annual variability* We have found that the inter-annual variability of GRACE can be only partly related to anomalies in precipitation and temperature, confirming the results of previous regional studies. Although limitations of the considered atmospheric reanalysis may alter the results at the regional level, this suggests that the inter-annual variability of GRACE is only partly related to the investigated atmospheric drivers, potentially highlighting the role of human water use as additional driver.
- *Seasonal variability* We have provided a comprehensive overview on the seasonality of terrestrial water storage and related it to the seasonal cycles of both precipitation and temperature. In tropical and equatorial regions, the seasonal cycle of precipitation was generally found to precede terrestrial water storage with a temporal lag of one to 2 months, while the seasonal cycle of temperature would typically be phase shifted by 6 months with respect to water storage. However, this was clearly not the case in temperate and cold regions, which is probably due to the more complex interplay between precipitation, storage processes, snow dynamics and temperature.
- *Subseasonal variability* We have shown that high-frequency variability of the GRACE record can be reconstructed from precipitation anomalies once an adequate averaging filter is applied to the daily precipitation forcing. This filter was designed to explicitly take the effect of earlier precipitation into account when comparing daily precipitation series with monthly GRACE data. This new method yields substantially better results compared to the classical approach based on monthly arithmetic means, providing a new perspective on the hydrological value of subseasonal (month to month) fluctuations of the GRACE signal, which have partly been interpreted as noise in previous studies.
- *Droughts* Finally, we have surveyed extreme dry events in the GRACE time series. The most important anomalies in terms of water storage deficits were documented on a global scale and related to droughts already described in the existing literature. Undocumented features were also identified using this global approach and will be subject to further investigation.

In summary, we have surveyed key features of temporal variability in the GRACE record and related them to the dominant atmospheric drivers, in contrast to the common practice of comparing GRACE terrestrial water storage to hydrological model simulations. We have related our results to physical interpretations from the rich body of regional GRACE studies, resulting in a comprehensive overview which will both contribute to a general understanding of terrestrial water storage and provide a global observation-based reference for hydrologists and climate scientists. As a novelty, we have shown that high-frequency (month to month) fluctuations of the GRACE signal contain a meaningful hydrological signal, which can be reconstructed from daily precipitation forcing. These findings have important implications for the assessment of GRACE uncertainties as well as for comparisons with hydrological model simulations.

## Appendix 1: STL for Unevenly Spaced Time Series

STL was introduced by Cleveland et al. (1990) for evenly spaced time series. In this appendix, we present a summary of a simplified STL procedure that includes a modification of the original algorithm for unevenly spaced time series. An important difference with the original STL authored by Cleveland et al. is that the seasonal cycle is defined as constant, i.e. it is not allowed to vary in shape or amplitude over time. We also introduce a modification in the computation of robustness weights that accounts for time series exhibiting large differences in variance (heteroskedasticity) between seasons.

Our focus is to decompose the total signal of a given time series ${\mathbf{X}} = \left\{ {x_{1} , \ldots ,x_{i} , \ldots ,x_{n} } \right\}$ associated with a time vector ${\mathbf{T}} = \left\{ {t_{1} , \ldots ,t_{i} , \ldots ,t_{n} } \right\}$ into a constantly repeating seasonal component *X*_seas_, a long-term trend component *X*_long_ and the remaining subseasonal residuals *X*_sub_.

The STL procedure consists of an inner loop and an outer loop. In the inner loop, seasonal and trend components are estimated from the time series using several passes of smoothing filters. In the outer loop, robustness weights are estimated to reduce the influence of outliers in the time series and are used in the next iteration of the inner loop. This procedure stops once some user-defined stability criterion has been reached.

### Locally Weighted Regression (Loess)

Since the algorithm involves multiple passes of a smoothing filter based on locally weighted regression (Loess), we first present a generic formulation for this smoothing filter. The Loess estimator of $x$ at time $t_{z}$ is denoted $g\left( {t_{z} } \right)$ and is given by a weighted polynomial fit of degree *d* to the values of **X** that are in the vicinity of $t_{z}$ and are given some weights $w$. This vicinity is restricted in time by a maximum time-lag parameter $\lambda$. The weights $w_{i}$ associated with each values of **X** that are in the vicinity of $t_{z}$ are given by the tricube function (Eq. 11).

11 $$w_{i} = \left\{ {\begin{array}{*{20}c} {0,\quad {\text{if}} \quad \left| {D\left( {t_{i} , t_{z} } \right)} \right| > \lambda } \\ {\left[ {1 - \left( {\frac{{\left| {D\left( {t_{i} ,t_{z} } \right)} \right|}}{\lambda }} \right)^{3} } \right]^{3} , \quad {\text{if}} \quad \left| {D\left( {t_{i} , t_{z} } \right)} \right| \le \lambda } \\ \end{array} } \right.$$

where $D\left( {t_{i} , t_{z} } \right)$ is a function of the distance in time between the two points. The values of **X** closest to $t_{z}$ will thus have the highest weights. Hence, for each prediction point $t_{z}$, **X** is associated with a vector of weights **W** that depends on $t_{z}$, $\lambda$ and the nature of the distance function $D$:

12 $${\mathbf{W}}\left( {t_{z} ,\lambda ,D} \right) = \left\{ {w_{i} , \ldots ,w_{n} } \right\}$$

The degree of the polynomial fit $g\left( {t_{z} } \right)$ that is fitted at each $t_{z}$ has to be chosen by the user depending on the application (usually a degree 1 or 2 polynomial is sufficient). Once this is performed for every time step in **T**, we obtain **G** = $\left\{ {g\left( {t_{1} } \right), \ldots ,g\left( {t_{n} } \right)} \right\}$, the so-called *local regression* of the original time series.

We introduce two different metrics for the distance $D\left( {t_{i} , t_{z} } \right)$ between two points in time. The first one is simply an *absolute distance* in time and is given by:

13 $$D_{\text{abs}} \left( {t_{i} , t_{z} } \right) = t_{i} - t_{z}$$

The second one is the *periodic distance* and depends on the length *p* of the chosen periodicity, for instance, suppose the time vector **T** is in units of days, then *p* will be equal to 365.25 days for a seasonal periodicity. The periodic distance is given by:

14 $$D_{\text{per}} \left( {t_{i} , t_{z} } \right) = \left[ {\left( {t_{i} - t_{z} + \frac{p}{2}} \right)\bmod p} \right] - \frac{p}{2}$$

where mod is the modulo operator. Thus, time points spaced from $t_{z}$ by multiples of exactly *p* days are assigned a null distance, and then, the distance linearly increases until the time points spaced by multiples of *p/2* days are assigned the maximum distance. Note that these distance metrics are used not only for the calculation of weights but also as the input vector of the polynomial fit $g\left( {t_{z} } \right)$ to the values of **X**.

### Inner Loop

The inner loop of the STL procedure aims at estimating the seasonal and long-term components of the time series **X** through the following steps.

#### *Step 1 Compute the detrended time series****X****

On the very first pass of the inner loop, the long-term trend ***X***_long_ will not be known yet so that **X*** is simply the original time series **X**. Otherwise, ***X***_long_ is subtracted from **X**, yielding the detrended time series **X***.

#### *Step 2 Compute the seasonal cycle***X**_seas_

The seasonal cycle is estimated at all points in **T** from **X*** using Loess with $D_{\text{per}}$ and a free parameter $\lambda_{\text{per}}$ that defines the vicinity in terms of periodic distance. The weight vector used in the local polynomial regression is denoted $W_{\text{per}}$:

15 $$W_{\text{per}} \left( {t_{z} } \right) = W\left( {t_{z} ,\lambda_{\text{per}} ,D_{\text{per}} } \right)$$

Note: after the first iteration of the inner and outer loops, $W_{\text{per}}$ is used in combination with the robustness weights $W_{\text{robust}}$. The weights simply correspond to the product of $W_{\text{per}}$ and $W_{\text{robust}}$.

#### *Step 3 Compute deseasonalized time series****X***^D^

A deseasonalized time series **X**^D^ is obtained by subtracting the seasonal cycle ***X***_seas_ from the original time series **X**.

#### *Step 4 Compute the long*-*term trend X*_long_

The long-term trend is estimated after applying Loess to the deseasonalized time series **X**^D^ with the distance function $D_{\text{abs}}$ and parameter $\lambda_{\text{long}}$ which defines the associated weight vector $W_{\text{long}}$.

16 $$W_{\text{long}} \left( {t_{z} } \right) = W\left( {t_{z} ,\lambda_{\text{long}} ,D_{\text{abs}} } \right)$$

As in step 2, these weights are used in combination with the robustness weights $W_{\text{robust}}$ after the first iteration of the inner and the outer loop.

### Outer Loop

Once an initial run of the inner loop has been carried out, the original time series can be decomposed into **X** = ***X***_long_ + ***X***_seas_ + **X**_resid_, where **X**_resid_ corresponds to the residuals. Following Cleveland et al. (1990), extremely large residuals are assumed to correspond to outliers and are assigned a small or a zero weight. These weights are defined using the bisquare function:

17 $$w_{\text{robust}} = \left\{ {\begin{array}{*{20}c} {0, \quad {\text{if}}\quad \left| {x_{\text{resid}} } \right| > h} \\ {\left[ {1 - \left( {\frac{{\left| {x_{\text{resid}} } \right|}}{h}} \right)^{2} } \right]^{2} , \quad {\text{if}}\quad \left| {x_{\text{resid}} } \right| \le h} \\ \end{array} } \right.$$

where *h* is:

18 $$h = 6 \cdot {\text{median}}\left( {\left| {{\mathbf{X}}_{\text{resid}} } \right|} \right)$$

However, a problem arises when time series exhibit seasonal heteroskedasticity because the value of *h* would change when different seasons of the time series are considered. A typical case of seasonal heteroskedasticity is when precipitation totals are very low during the dry season but exhibit high variability during the wet season. If we followed the approach of Cleveland et al., relatively small outliers in the dry season would be unlikely detected, whereas large but still realistic variations during the wet season would be more likely detected as outliers. This problem can be avoided by introducing seasonally varying estimates of *h*. This is done by calculating *h* at each prediction point $t_{z}$ using a weighted median with the weights $W_{\text{per}}$ computed from the periodic distance (step 2):

19 $$h\left( {t_{z} } \right) = 6 \cdot {\text{median}}\left( {\left| {{\mathbf{X}}_{\rm resid} } \right|,W_{\rm per} \left( {t_{z} } \right)} \right)$$

### Choosing the Parameters

The following parameters need to be defined: *p* the length of each cycle of the seasonal component; *d* the degree of the weighted polynomial regression; n_inner_ the number of passes through the inner loop; n_outer_ the number of iterations of the outer loop; $\lambda_{\text{per}}$ the maximum time lag for the computation of the seasonal component; $\lambda_{\text{long}}$ the maximum time lag for the computation of the long-term component

Parameter *p* is obviously determined by the nature of the investigated time series (here,* p* = 365.25). We chose a polynomial of degree *d* = 2 in order to hinder smoothing of the low and high peaks of the seasonal cycle. For the trend component, a polynomial of degree *d* = 1 is sufficient. The number of passes and iterations was chosen so that the resulting decomposition reaches stability. For the number of passes of the inner loop, Cleveland et al. recommend *n*_inner_ = 2. Regarding *n*_outer_, we experimentally determined that *n*_outer_ = 3 was sufficient for our application.

The parameter $\lambda_{\text{per}}$ determines the smoothness of the seasonal cycle, with larger values, resulting in a smoother estimate of the seasonal cycle. On the other hand, smaller values of $\lambda_{\text{per}}$ will reduce the number of points actually used in the local regression so that the resulting seasonal cycle is more likely to be affected by outliers or sudden changes arising from the uneven spacing of the time series. Hence, the choice of $\lambda_{\text{per}}$ is a balanced consideration between accuracy and robustness in the representation of the seasonal cycle. In this paper, a good compromise was experimentally found with $\lambda_{\text{per}} = 60$ days.

The parameter $\lambda_{\text{long}}$ controls the degree of leakage of the long-term component into the residuals. Larger values of the parameter will result in a smoother estimate of the trend but also cause some of the long-term signal to be incorporated into the residuals. Vice versa, smaller values of this parameter will make the long-term component more sensitive to high-frequency variability. In this application, we followed the recommendations from Cleveland et al. who showed that $\lambda_{\text{long}}$ = 1.5 × *p* provides a good compromise in most cases.

## Appendix 2

### Analytical Integration of the Weighting Function

Integrating Eq. 6 over the interval $\left[ {a_{j} ,b_{j} } \right]$ associated with the monthly interval $t_{j}^{*}$ must be done with care since the function $w\left( {t,t_{i} } \right)$ is discontinuous. In total, three cases can be considered: (1) the continuous case where $a_{j} \ge t_{i}$ (Fig. 4a), (2) the discontinuous case where $a_{j} < t_{i} \le b_{j}$ (Fig. 4b) and (3) the continuous case where $b_{j} < t_{i}$. For convenience, we note $a_{j} = a$ and $b_{j} = b$:

20 $$\begin{aligned} W\left( {t_{j}^{*} ,t_{i} } \right) & = \mathop \int \limits_{a}^{b} w\left( {t,t_{i} } \right) {\text{d}}t \\ & = \left\{ {\begin{array}{*{20}l}{\mathop \int \limits_{a}^{b} w\left( {t,t_{i} } \right) {\text{d}}t}&{ \quad {\text{if}} \quad a \ge t_{i} } \\ {\mathop \int \limits_{a}^{{t_{i} }} w\left( {t,t_{i} } \right) {\text{d}}t + \mathop \int \limits_{{t_{i} }}^{b} w\left( {t,t_{i} } \right) {\text{d}}t}&{ \quad {\text{if}} \quad a < t_{i} \le b} \\ {\mathop \int \limits_{a}^{b} w\left( {t,t_{i} } \right) {\text{d}}t}&{ \quad {\text{if}} \quad b < t_{i} } \\ \end{array} } \right. \\ & = \left\{ {\begin{array}{*{20}l} { - \tau {\text{e}}^{{ - \frac{1}{\tau }\left( {b - t_{i} } \right)}} + \tau {\text{e}}^{{ - \frac{1}{\tau }\left( {a - t_{i} } \right)}} }&{ \quad {\text{if}} \quad a \ge t_{i} } \\ { - \tau {\text{e}}^{{ - \frac{1}{\tau }\left( {b - t_{i} } \right)}} + \tau }&{ \quad {\text{if}} \quad a < t_{i} \le b} \\ {0}&{ \quad {\text{if}} \quad b < t_{i} } \\ \end{array} } \right. \\ \end{aligned}$$

The normalized version of $W\left( {t_{j}^{*} ,t_{i} } \right)$ is given by (equivalently to Eq. 8):

21 $$\hat{W}\left( {t_{j}^{*} ,t_{i} } \right) = \frac{{W\left( {t_{j}^{*} ,t_{i} } \right)}}{{\mathop \int \nolimits_{ - \infty }^{ + \infty } W\left( {t_{j}^{*} ,t_{i} } \right) {\text{d}}t_{i} }}$$

The denominator has to be decomposed into three continuous parts:

22 $$\mathop \int \limits_{ - \infty }^{ + \infty } W\left( {t_{j}^{*} ,t_{i} } \right) {\text{d}}t_{i} = \mathop \int \limits_{ - \infty }^{a} W\left( {t_{j}^{*} ,t_{i} } \right) {\text{d}}t_{i} + \mathop \int \limits_{a}^{b} W\left( {t_{j}^{*} ,t_{i} } \right) {\text{d}}t_{i} + \mathop \int \limits_{b}^{ + \infty } W\left( {t_{j}^{*} ,t_{i} } \right) {\text{d}}t_{i}$$

The first part yields:

23 $$\begin{aligned} \mathop \int \limits_{ - \infty }^{a} W\left( {t_{j}^{*} ,t_{i} } \right) {\text{d}}t_{i} & = \left( {\tau^{2} {\text{e}}^{{ - \frac{1}{\tau }\left( {b - a} \right)}} - \tau^{2} {\text{e}}^{{ - \frac{1}{\tau }\left( {a - a} \right)}} } \right) - \left( {\tau^{2} {\text{e}}^{{ - \frac{1}{\tau }\left( {b + \infty } \right)}} - \tau^{2} {\text{e}}^{{ - \frac{1}{\tau }\left( {a + \infty } \right)}} } \right) \\ & = \tau^{2} {\text{e}}^{{ - \frac{1}{\tau }\left( {b - a} \right)}} - \tau^{2} \\ \end{aligned}$$

The second part yields:

24 $$\begin{aligned} \mathop \int \limits_{a}^{b} W\left( {t_{j}^{*} ,t_{i} } \right) {\text{d}}t_{i} & = \left( {\tau^{2} {\text{e}}^{{ - \frac{1}{\tau }\left( {b - b} \right)}} + \tau b} \right) - \left( {\tau^{2} {\text{e}}^{{ - \frac{1}{\tau }\left( {b - a} \right)}} + \tau a} \right) \\ & = \tau^{2} + \tau b - \tau^{2} {\text{e}}^{{ - \frac{1}{\tau }\left( {b - a} \right)}} - \tau a \\ \end{aligned}$$

And the third part is:

25 $$\mathop \int \limits_{b}^{ + \infty } W\left( {t_{j}^{*} ,t_{i} } \right) {\text{d}}t_{i} = 0$$

Thus after combining equations 23, 24 and 25, equation 22 can be rewritten as:

26 $$\begin{aligned} \mathop \int \limits_{ - \infty }^{ + \infty } W\left( {t_{j}^{*} ,t_{i} } \right) {\text{d}}t_{i} & = \tau^{2} {\text{e}}^{{ - \frac{1}{\tau }\left( {b - a} \right)}} - \tau^{2} + \tau^{2} + \tau b - \tau^{2} {\text{e}}^{{ - \frac{1}{\tau }\left( {b - a} \right)}} - \tau a + 0 \\ & = \tau \left( {b - a} \right) \\ \end{aligned}$$

which is then injected in Eq. 21, yielding the normalized weighting function (Eq. 9):

27 $$\begin{aligned}\hat{W}\left( {t_{j}^{*} ,t_{i} } \right) = \frac{{W\left( {t_{j}^{*} ,t_{i} } \right)}}{{\mathop \int \nolimits_{ - \infty }^{ + \infty } W\left( {t_{j}^{*} ,t_{i} } \right) {\text{d}}t_{i} }} &= \left\{ {\begin{array}{*{20}l} {\frac{{{\text{e}}^{{ - \frac{1}{\tau }\left( {b - t_{i} } \right)}} + e^{{ - \frac{1}{\tau }\left( {a - t_{i} } \right)}} }}{b - a}}&{ \quad {\text{if}}\quad a \ge t_{i} } \\ {\frac{{{\text{e}}^{{ - \frac{1}{\tau }\left( {b - t_{i} } \right)}} + 1}}{b - a}}&{ \quad {\text{if}}\quad a < t_{i} \le b} \\ {0}&{ \quad {\text{if}}\quad b < t_{i} } \\ \end{array} } \right. \\ &= \left\{ {\begin{array}{*{20}l} {\frac{{{\text{e}}^{{\frac{1}{\tau }\left( {t_{i} - a} \right)}} - e^{{\frac{1}{\tau }\left( {t_{i} - b} \right)}} }}{b - a}}&{ \quad {\text{if}}\quad a \ge t_{i} } \\ {\frac{{1 - {\text{e}}^{{\frac{1}{\tau }\left( {t_{i} - b} \right)}} }}{b - a}}&{ \quad {\text{if}}\quad a < t_{i} \le b} \\ {0}&{ \quad {\text{if}}\quad b < t_{i} } \\ \end{array} } \right.\end{aligned}$$
